# Biochemical characterization of a recombinant laccase from *Halalkalibacterium halodurans* C-125 and its application in the biotransformation of organic compounds

**DOI:** 10.1007/s10529-024-03532-w

**Published:** 2024-10-28

**Authors:** Jihene Maati, Jolanta Polak, Monika Janczarek, Marcin Grąz, Issam Smaali, Anna Jarosz-Wilkołazka

**Affiliations:** 1 Laboratory of Protein Engineering and Bioactive Molecules (LIP-MB-LR11ES24), National Institute of Applied Sciences and Technology INSAT-BP 676, University of Carthage, 1080 Tunis Cedex, Tunisia; 2 Department of Biochemistry and Biotechnology, Institute of Biological Sciences, Maria Curie-Skłodowska University, Akademicka 19, 20-033 Lublin, Poland; 3 Department of Industrial and Environmental Microbiology, Institute of Biological Sciences, Maria Curie-Skłodowska University, Akademicka 19, 20-033 Lublin, Poland

**Keywords:** Biotransformation, Green catalyst, *Halalkalibacterium halodurans*, Laccase

## Abstract

**Objectives:**

This study aimed to produce an engineered recombinant laccase from extremophilic *Halalkalibacterium halodurans* C-125 (Lac-*HhC-125)* with higher protein yield, into a more active conformation and with properties that meet the fundamental needs of biotechnological application.

**Results:**

The rLac-*HhC125* was partially purified by size exclusion chromatography and concentrated by ultrafiltration (10 kDa) with a yield of 57.6%. Oxidation reactions showed that adding 2 mM CuSO_4_ to the assay solution led to activating the laccase. To increase its initial activity, the rLac-*HhC125* was treated at 50 °C for 20 min before the assays, improving its performance by fourfold using the syringaldazine as a substrate. When treated with EDTA, methanol, ethanol, and DMSO, the rLac-*HhC125* maintained more than 80% of its original activity. Interestingly, the acetonitrile induced a twofold activity of the rLac-*HhC125*. The putative rLac-*HhC125* demonstrated a capability of efficient transformation of different organic compounds at pH 6, known as dye precursors, into coloured molecules.

**Conclusion:**

The rLac-*HhC125* was active at high temperatures and alkaline pH, exhibited tolerance to organic solvents, and efficiently transformed different hydroxy derivatives into coloured compounds, which indicates that it can be used in various biotechnological processes.

**Supplementary Information:**

The online version contains supplementary material available at 10.1007/s10529-024-03532-w.

## Introduction

Oxidation processes using organic and inorganic oxidants as well as conventional chemical catalysts have impacts on the environment and human health (Kumari and Kumar 2023). Enzymes are regarded as green oxidative catalysts, as enzyme catalysis occurs in mild conditions without the use of toxic reagents. Enzymatic catalysis with laccases (EC 1.10.3.2, p-diphenol: dioxygen oxidoreductase) fulfils the basic requirement of green chemistry, in which milder conditions are required and little toxic waste is produced (Romero-Guido et al. 2018). The basic laccase reaction mechanism involves the use of molecular oxygen as the final electron receptor and the release of two water molecules as a by-product. The redox mechanism occurs due to the presence of four copper atoms (T1Cu, T2Cu, T3aCu, T3bCu) (Paraschiv et al. 2022).

Laccases are very unique and versatile enzymes reported to be present in plants, insects, bacteria, and fungi (Janusz et al. 2020). Fungal laccases exhibit high redox potential; they are secreted extracellularly at high levels, and their application has attracted considerable attention in recent years, for instance in the biodegradation of synthetic dyes (HajKacem et al. 2020), wastewater treatments (Haj Kacem et al. 2018), and the synthesis of new molecules, such as dyes with bioactive properties (Polak et al. 2022). Bacteria that synthesize laccase represent the phyla Firmicutes—*Bacillus* (Hahn 2023)*, Geobacillus* (Basheer et al. 2017)*, Lysinibacillus* (Ouyang et al. 2022)*, Aquisalibacillus* (Rezaei et al. 2017)*, Staphylococcus* (Li et al. 2020)*,* and *Rhodococcus* (Santo et al. 2013)*,* Actinobacteria—*Streptomyces* (Fernandes et al. 2014)*,* Euryarchaeota—*Haloferax* (Kasirajan et al. 2021)*,* Deinococcota—*Thermus* (Kumari et al. 2018)*,* and Proteobacteria—*Escherichia* (Ihssen et al. 2015)*, Pseudomonas* (Neifar et al. 2016)*, Sinorhizobium* (Pawlik et al. 2016)*,* β*-proteobacterium* (Kumar et al. 2015), *Yiersinia* (Singh et al. 2016), *Klebsiella* (Liu et al. 2017)*,* and *Enterobacter* (DeAngelis et al. 2013). *Bacillus* laccases possess many attractive properties including activity in a wide pH value range, thermal stability, and resistance to organic solvents, metal ions, and inhibitors, which are required in bio-bleaching (Sondhi et al. 2015), biorefinery (Cheng et al. 2021), bioremediation (Sun et al. 2017) and food dye decolourization (Li et al. 2022). *Bacillus* laccases can also potentially be used as biosensors (Zhao et al. 2023) and anti-proliferative agents against cancer cells (Sondhi et al. 2021).

*Halalkalibacterium* is a Firmicutes genus in the family Bacillaceae. *Halalkalibacterium halodurans* bacteria live in alkaline and hypersaline conditions (Takami et al. 2000), which makes the species interesting host-producing enzymes. *H. halodurans* C-125 (previously known as *Bacillus halodurans* C-125) with a whole-sequenced genome has been shown to produce lignocellulosic enzymes (Takami et al. 2000). Many of these enzymes have been proved to be active at high temperature and pH values (Akita et al. 2005; Smaali et al. 2006; Zeeshan et al. 2018; Mahmood et al. 2019).

In the present study, a gene encoding a putative laccase from *Halalkalibacterium halodurans* C-125 was cloned within *E. coli* BL21 (DE3), resulting in a recombinant version of the enzyme. The recombinant laccase was expressed as a protein lacking the native signal peptide sequence for the intracellular expression of the protein and designated as rLac-*HhC125*. The rLac-*HhC125* was partially purified, biochemically characterized, and applied for the transformation of organic compounds belonging to methoxy-, hydroxy-, and amino-derivatives as precursors for dye synthesis. To the best of our knowledge, there are no reports on studies of laccase from *H. halodurans* C-125 and its usability in the transformation of different organic compounds into valuable products, such as dyes.

## Material and methods

### Strains, plasmid, and chemicals

*Halalkalibacterium halodurans* C-125, referenced in the NCBI taxonomy database as NCBI: txid272558, was obtained from the Japan Collection of Microorganisms (JCM9153) and used in this study as a source of the gene encoding the enzyme of interest (Smaali et al. 2006). *Escherichia coli* strains DH5α and BL21(DE3) (Thermo-Scientific, Waltham, Massachusetts, USA) were used for gene cloning and protein expression, respectively. PCR products were purified using a clean-up kit from A&A Biotechnology (Poland). Restriction enzymes were purchased from Thermo-Fisher Scientific. Plasmid pET28-a ( +) (Novagen, Darmstadt, Germany) was isolated from *E. coli* using the Miniprep Express™ solution (MP Biomedicals, USA). Kanamycin, isopropyl-β-D-thiogalactoside (IPTG), REDTaq® ReadyMix™, 1 kb DNA ladder (GeneRuler). Mix ready to use, and lysozyme were purchased from Sigma. SimplySafe DNA gel loading buffer (EURx, Poland) was used for analysis of PCR amplicons in gel electrophoresis. *Cerrena unicolor* laccase was obtained and purified according to a previously described method (Polak et al. 2022).

Organic compounds were purchased from Sigma-Aldrich-Fluka Company (currently Merck). These included syringaldazine (SGZ), guaiacol, 5-amino-2-hydroxybenzoic acid (5-aminosalicylic acid; 5A2HBA), 4-amino-2-hydroxybenzoic acid (4A2HBA), 3-amino-4-hydroksybenzoic acid (3A4HBA), 3-amino-4-hydroxybenzenesulfonic acid (AHBS), catechol (Cat), 4-methoxyphenol (4MxPh), 4,5-dihydroxy-1,3-benzene-disulfonic acid (dHBdSA), 6-hydroxy-2-naphthalenesulfonic acid sodium salt hydrate (HNSA), 4-amino-3-hydroxy-1-naphthalenesulfonic acid (4AHNS), 6-amino-4-hydroxy-2-naphthalenesulfonic acid (6AHNS), 7-amino-4-hydroxy-2-naphthalene sulfonic acid (7AHNS), 4-amino-5-hydroxynaphthalene-2,7-disulfonic acid monosodium salt hydrate (4A5HNDSA), 3-(3,4-dihydroxyphenyl)-L-alanine (DHPAla), 2-amino-3-hydroxypyridine (2A3HP), 4-amine-3-hydroxy-1-naphthalene sulfonic acid (4AHNS), and D-catechin (CatN). Most of the substrates were dissolved in water or water with small addition of NaOH, except for SGZ, which was dissolved in 95% ethanol.

### Sequence analysis and gene amplification

The presence of a native signal peptide sequence was predicted using the SignalP 5.0 tool (SignalP—5.0—Services—DTU Health Tech). The Blast program (BLAST: Basic Local Alignment Search Tool; nih.gov) was used for nucleotide sequence similarity search with the standard program default. Multiple sequence alignment and analysis of the data were performed using the ClustalW tool and the phylogenetic tree was built with the software package MEGA version 11.0 (megasoftware.net) using the Neighbor-Joining method and the bootstrap parameter model of distance analysis. The topology of the resultant tree was evaluated by the bootstrap analysis with 1000 replicates. The protein structure of the *H. halodurans* C-125 laccase was predicted with the online I-TASSER server (I-TASSER server for protein structure and function prediction (zhanggroup.org) (Yang et al. 2015). The 3-D structure was analyzed and edited using PyMol software.

To amplify the laccase gene without the native signal peptide by PCR, two primers were designated and synthesized according to a sequence from *H. halodurans* C-125 available at the NCBI database (GenBank accession number: BAB05801). The laccase gene was amplified by PCR using genomic DNA of *H. halodurans* C-125 as a template and Jump start RED Taq Mix (Sigma) in a thermal cycler (Biometra T, Germany). The standard reaction conditions were 3 min at 95 °C, followed by 35 cycles of 30 s at 95 °C, 50 s at 56.8 °C, 2 min at 72 °C, and finally 10 min at 72 °C. The **forward primer (**5'-CCGGCGGATCC**ATG**GACGATCATTCTGAAATGGATC-3') was designed around the start codon (bold face) of open reading frame (ORF) BH2082 of *H. halodurans* C-125 and contained an introduced *Bam*HI site (underlined) for cloning purposes. The **reverse primer** (5'-CCGGCAAGCTT**TTA**CTCAGGCATATTTGGAATATTAGG-3') was designed on the reverse complement strand around the TAA stop codon (bold face) of ORFbh2082, with an introduced *Hind* III site (underlined). The *Bam*HI and *Hind*III restriction sites were incorporated in the primer sequence to allow *in-frame* cloning of the PCR product into the corresponding restriction sites located in the polylinker of the pET28 (a) + plasmid. Both expression vector pET28-a ( +) and the PCR products were digested with *Bam*HI and *Hind*III enzymes (Thermo Fisher Scientific, USA). The amplified gene after digestion by *Bam*HI and *Hind* III was cloned into the *Bam*HI/*Hind*III-digested vector pET28-a ( +). Chemically competent DH5-α cells were transformed with the ligation mixture. The mass of insert required at molar insert vector ratios (insert: vector) was calculated with NEBioCalculator software (NEBioCalculator) to optimize the typical ligation reaction. Screening of positive clones was carried out by colony PCR and plasmid sequencing. Finally, a positive clone was obtained, which contained the proper construct of the laccase gene cloned in a respective reading frame in vector pET28-a ( +) (named as pET28-a( +)/*Lac*). Next, the DNA of recombinant plasmid pET28-a ( +)/*Lac* was isolated from strain DH5-α and used for transformation of *E. coli* BL21(DE3) cells for recombinant protein expression.

### Heterologous expression and partial purification

One colony of *E. coli* strain BL21(DE3) carrying pET28-a ( +)/*Lac* with the laccase gene from *H. halodurans* C-125 was inoculated in Luria Bertani (LB) broth containing 30 µg kanamycin mL^−1^ and grown for 16 h at 37 °C. The overnight preculture was diluted (1:100) in Terrific Broth (TB) medium containing 30 µg kanamycin mL^−1^, and the cultures obtained were grown in the same conditions until reaching an optical density OD_600_ = 0.4–0.5. Then, they were used for determination of the efficiency of laccase gene expression by testing different parameters, such as IPTG concentration (0.5–1 mM), Cu^2+^ concentration (0–2 mM), induction temperature (19–28 °C), and shaker agitation (190–250 rpm). For purification of the expressed laccase, the induced cultures were centrifuged at 8000 × *g* for 10 min at 4 °C, and the pellets were washed 3 times with 50 mM sodium phosphate buffer (pH 7) to remove the rest of the medium components. Next, the washed pellets were resuspended in cold sodium phosphate lysis buffer (50 mM, pH 7) containing 10% glycerol and 1 pallet of the cocktail mini EDTA-free antiproteases (Roche). Resuspended cells were sonicated on ice at 50% amplitude for 5 min with 10 s ON/10 s OFF pulses. Disrupted cells were removed by centrifugation (20,000 × *g*, 40 min, 4 °C), and the supernatant was used for further purification steps.

Chromatographic separation was performed using a BioRad BioLogic LP chromatograph. The laccase was partially purified in a Sephadex G-100 column with a flow rate of 0.25 mL min^−1^. Elution was performed using 0.02 M sodium phosphate buffer pH 7. The Sephadex G-100 was calibrated with protein standards (GE Healthcare, Sweden): ovalbumin (43 kDa), conalbumin (75 kDa), and aldolase (158 kDa). The eluate was collected using a fraction collector (Bio-Rad, USA). The volume of each fraction was 1 mL. The laccase activity in the protein-rich fractions was determined using SGZ as a substrate. The laccase-rich fractions were pooled together, concentrated through ultrafiltration (membrane 10,000 kDa MWCO, Sartorius), and stored at −20 °C until further use.

The total protein concentration in pooled fractions was determined according to the Bradford method (Bradford 1976). A calibration curve was prepared using bovine serum albumin as a standard, and the concentration was expressed in micrograms per milliliter (µg mL^−1^).

### Gel electrophoresis and zymography

SDS-PAGE of the obtained laccase was performed according to the method proposed by (Laemmli 1970) using 12% running gel and 5% stacking gel of polyacrylamide. Electrophoresis was performed at 120 V. The proteins were visualized by gel staining with Coomassie Brilliant Blue R-250 (Bio-Rad). For molecular mass determination, the PageRuler Prestained Protein Ladder (Thermo-Scientific) was used as a protein standard.

For laccase activity staining, the gel was washed with 12.5% Triton-X 100 to remove the SDS and renature the protein. After three washing steps, the Triton-X 100 was removed, and the gel was allowed to stand in a bath containing 100 mM Tris–HCl buffer (pH 8) and 2 mM CuSO_4_ at 40 °C. After 15 min of preincubation, 50 µL of guaiacol was added, and the laccase activity characterized by a yellowish-brown colour was visualized in the gel after approximately 5 to 10 min of incubation.

### HPLC analysis

High-performance liquid chromatographic analyses were performed using an Agilent 1260 Infinity (Agilent®, Germany) chromatograph coupled with a diode array detector. The molecular mass of the laccase was monitored by high-performance size exclusion chromatography (Phenomenex, Yarra 1.8 µm, SEC-X150 column 150 × 4.6 mm). Isocratic elution was carried out using Milli-Q water. The total run time of each analysis was 20 min. After each analysis, a 5-min post-run was conducted to restore the start conditions of the analysis. The eluent flow rate was kept at 0.2 mL min^−1^ throughout the separation process, and the separation column temperature was maintained at 20 °C. Each 2-µL sample was injected using an autosampler. The elution of compounds was monitored at a wavelength of 280 nm. Agilent OpenLAB CDS ChemStation LC and CE Drivers (A.02.10 (026) version) software was used for data processing and reporting. Identification of the protein peak was achieved by comparing retention times with the standard of protein of known molecular mass (aldolase 158 kDa, conalbumin 75 kDa, ovalbumin 43 kDa, carbonic anhydrase 29 kDa obtained from the GE Healthcare kit).

### Laccase activity assay

Laccase activity was determined spectrophotometrically by monitoring the oxidation of syringaldazine (SGZ) in the presence of 2 mM CuSO_4_. The oxidation of 0.025 mM SGZ in 100 mM Tris–HCl buffer (pH 8) was monitored at 530 nm (ε = 64,000 M^−1^ cm^−1^) (Ruijssenaars and Hartmans 2004). All measurements were done in triplicate along with duplicate measurements of autoxidation of all tested substrates with the buffer added instead of the enzyme. One unit of enzymatic activity (U) was defined as the amount of enzyme oxidizing 1 μmol of substrate per minute.

Simultaneously, control experiments under the possible oxidation of the SGZ by copper ions (CuSO_4_) were prepared; therefore, no laccase was added to the samples before the measurements.

### Biochemical and kinetic characteristics

To define the **optimal concentration of copper ions** used for a standard activity assay for the determination of recombinant laccase activity, pre-treatment of the laccase-buffer solution in the presence of different concentrations of CuSO_4_ (1–10 mM) was performed at 40 °C for 20 min. Afterwards, SGZ was added and the absorbance of its oxidation product was monitored at 530 nm. The laccase assay without the addition of CuSO_4_ was used as a control.

To define the **optimal temperature for the recombinant laccase pre-treatment step** for the standard activity assay, laccase was pre-treated in the temperature range of 25–70 °C for 20 min. The assay solution was composed of recombinant laccase, 100 mM Tris–HCl buffer (pH 8), and 2 mM CuSO_4_. After thermal pre-treatment, the mixture was cooled at room temperature, and the residual activity was determined by monitoring the oxidation of SGZ for 1 min. Laccase that was not subjected to the thermal-activation treatment and kept on ice was used as a control with a relative activity of 100%.

The effect of the **time of the recombinant laccase thermal-activation treatment** was investigated by treating the laccase at the selected temperature (50 °C) in different time intervals, and the activity was measured as described above. Laccase preincubated at 50 °C for 20 min was used as a control with a relative activity of 100%.

The **standard activity assay** for recombinant laccase included the step of its pre-treatment at 50 °C for 20 min in the presence of 2 mM CuSO_4_ in buffered conditions pH 8 before the addition of the substrate (SGZ). The oxidation of 0.025 mM SGZ in 100 mM Tris–HCl buffer (pH 8) was monitored at 530 nm (ε = 64,000 M^−1^ cm^−1^).

To determine **the optimal pH value for the activity assay** of the partially purified laccase, the substrate oxidation was assayed in reaction mixtures adjusted to various pH values between 3 and 10. The buffers used for this purpose were sodium acetate buffer (100 mM, pH 3–6), sodium phosphate buffer (100 mM, pH 7–7.5), Tris–HCl buffer (100 mM, pH 8–8.5), and glycine–NaOH buffer (pH 9–10). The effect of **pH values on the stability** of the partially purified laccase was investigated by measuring the remaining activity after laccase incubation in the buffer solutions mentioned above at 4 °C for 6 h. Maximum activity measured in the standard assay conditions was defined as the relative activity of 100%.

To determine **the optimal temperature for the activity assay** of the partially purified laccase, the enzyme was preincubated at selected temperatures from 20 °C to 90 °C in 100 mM Tris–HCl (pH 8), with a 10 °C interval during 20 min in the presence of 2 mM CuSO_4_. Afterward, SGZ was added to the sample, and oxidation was monitored at 530 nm. For the **thermal stability** analysis, the laccase was incubated for 180 min at a temperature of 30 °C, 40 °C, 50 °C, or 60 °C in Tris–HCl buffer (100 mM, pH 8) supplemented with 2 mM CuSO_4_. The assays of stability were conducted without prior thermal-activation treatment. Maximum activity was defined as the relative activity of 100%.

To test **the effects of metal ions** on laccase activity, Mg^2+^, Mn^2+^, Zn^2+^, and Fe^2+^ were added to the reaction mixture at a final concentration of 1 mM in two test variants. The first variant assessed the effect of the metal ions added to the reaction mixture before the thermal-activation treatment step and exposed to the standard assay conditions. The second variant determined the effect of the metal ions added to the reaction mixture containing recombinant laccase previously pre-treated at 50 °C for 20 min in the presence of 2 mM CuSO_4_ in buffered conditions pH 8. Afterward, SGZ was added and kinetic measurements were performed. A reaction mixture free of the laccase solution was used as a blank for the substitution of possible SGZ oxidation caused by metal ions. The SGZ oxidation in the standard laccase assay conditions was recorded as 100% (control).

**Inhibitors**, including EDTA, sodium azide, phenylthiourea, and L-cysteine, were added separately to the standard assay conditions with a final concentration of 1 mM, and the effect was determined during the oxidation of SGZ monitored for 1 min without a prior pre-treatment step. The effect of the investigated inhibitors on the laccase activity was also studied for laccase exposed to the thermal-activation step (50 °C, 20 min) of the standard assay conditions. The SGZ oxidation in the standard laccase assay conditions and without inhibitors was recorded as 100% (control).

**The effect of commonly used organic solvents,** including methanol, ethanol, acetonitrile, and DMSO, on laccase activity was checked in its final concentration of 10% (v/v), and the residual activity was measured for 1 min using SGZ as a substrate. The effect of organic solvents was studied for 20 min during the laccase pre-treatment step and for laccase previously pre-treated according to the standard assay conditions and without the presence of any organic solvent. The SGZ oxidation in the standard laccase assay conditions and the absence of organic solvents was recorded as 100% (control).

The kinetic constant *K*_M_ was determined using SGZ as a substrate in a series of concentrations ranging from 0.3 to 35 μM in the standard assay conditions. The laccase activity for the different substrate concentrations was determined in triplicate. The *K*_M_ value was evaluated by the Lineweaver–Burk plot.

### Biotransformation of organic compounds

Screened organic compounds (belonging to methoxy-, hydroxy-, and amino-derivatives) tested as potential dye precursors were used to evaluate the biotransformation ability of the partially purified recombinant laccase rLac-*HhC125*. The reaction mixtures contained 0.5 mM substrate, 100 mM sodium phosphate buffer (pH 6) or Tris–HCl buffer (pH 8), 2 mM CuSO_4_, and 10 U L^−1^ of rLac-*HhC125* final activity. The oxidation of substrates proceeded at 40 °C in shaking conditions (120 rpm) for 24 h. The ability of laccase to transform different organic compounds was determined spectrophotometrically as a relative increase in absorbance in the wavelength range between 380 and 800 nm. Purified laccase obtained from a fungal *Cerrena unicolor* strain (15 U L^−1^ final activity) was used for biotransformation control experiments, which were carried out at 26 °C for 24 h. All reactions were performed in triplicate.

## Results and discussion

### Laccase sequence analysis and amplification of the deduced gene

The *in-silico* analysis aimed to predict the characteristics of laccase from *Halalkalibacterium halodurans* C-125 based on a comparative analysis with putative laccases from bacteria and fungi. The corresponding amino acid sequences were retrieved from the Genbank database.

**Fig. 1:**
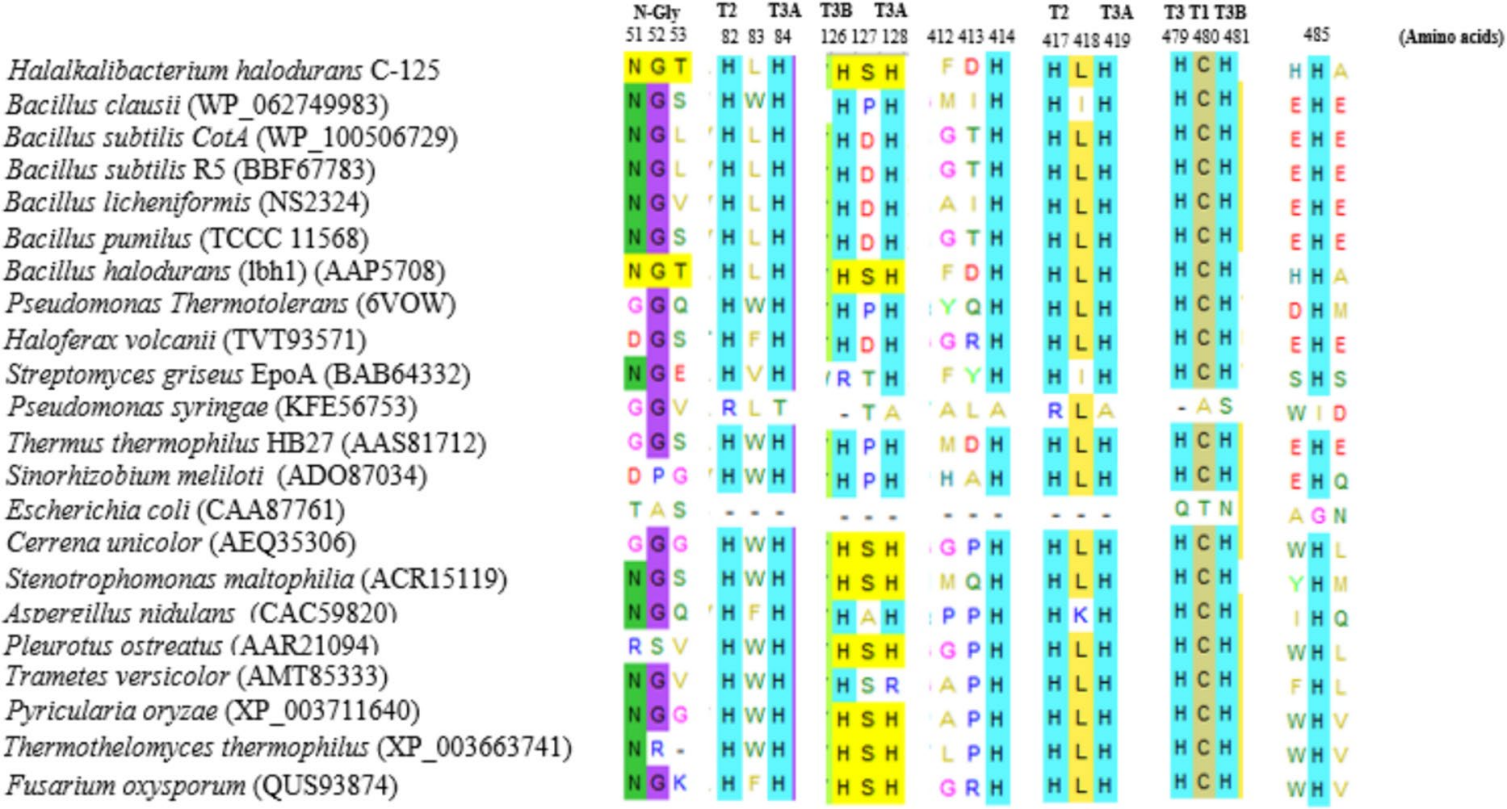
Amino acid sequence alignment of rLac-*HhC125* and other functional and characterized laccases from bacteria and fungi. The multiple sequence alignment was conducted using the MEGA V11.0 tool. T1, T2, T3A, and T3B indicate the putative corresponding type 1, 2, and 3 copper centers. *N*-Gly represents the glycosylation site of laccase

Figure 1 shows the multiple alignment of amino acid sequences derived from selected laccase origins. We observed a conserved H-C-H motif (His479-Cys 480-His 481) in the amino acid sequences of bacterial (*H. halodurans* C-125, *B. clausii*, *B. subtilis* (CotA), *B. subtilis* R5, *B. licheniformis*, *B. pumilus* TCCC 11568, *B. halodurans* (lbh1), *Pseudomonas thermotolerans*, *Haloferax volcanii*, *Streptomyces griseus* EpoA, *Thermus thermophilus* HB27, *Sinorhizobium meliloti*) and fungal (*Cerrena unicolor*, *Stenotrophomonas maltophilia* AAP56, *Aspergillus nidulans*, *Pleurotus ostreatus*, *Trametes versicolor*, *Pyricularia oryzae*, *Thermothelomyces thermophilus* ATCC 42464, *Fusarium oxysporum*) laccases suggesting the presence of a T1 copper site, which coordinates with three strong ligands (one cysteine and two histidine). The multiple alignment indicates the classification of rLac*HhC125* as a blue multicopper oxidase. By contrast, the *E. coli* CotA and *Pseudomonas syringae* laccase sequence lacks the H479-C480-H481 motif, suggesting that the corresponding laccases could be classified as white or yellow laccases.

**Fig. 2:**
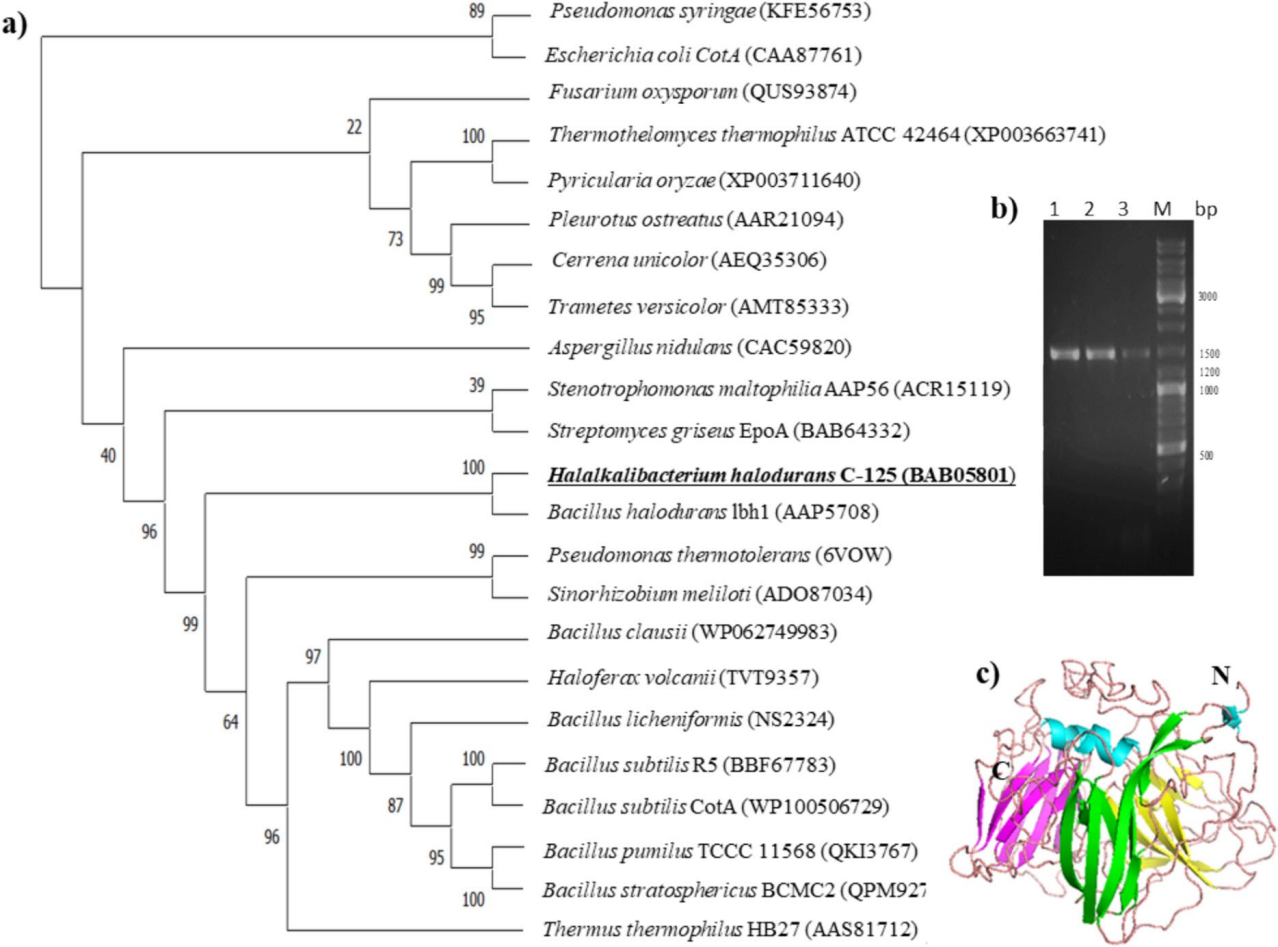
Dendrogram of rLac-*HhC125* and other characterized laccases from bacterial and fungal strains, showing phylogenetic analysis of the bases of the amino acid sequence (**a**); PCR amplification of the *H. halodurans* C-125 laccase deduced gene the predicted (**b**); 3-D homology model of rLac-*HhC125* using the I-TASSER tool (**c**). Alignment of amino acids was conducted by the ClustalW software. The phylogenetic tree was built using the MEGA version 11.0 tool. The I-Tasser model of the rLac-*HhC125* was predicted by PDB hit with its closest homologue multi-copper oxidase from *Pseudomonas thermotolerans* ‘PDB 6VOW’. The 3D structure was visualized and edited using the PyMol program. N and C are the N- and C- termini, respectively. The underlined species represents the source of laccase used in our study

In the phylogenetic tree presented in Fig. 2a, clusters containing sequences from the same ancestor are manifested as a grouping with a high level of confidence. The bacterial laccase sequences are grouped separately from the fungal proteins. The cluster with the sequences of *E. coli* CotA and *P. syringae* included exclusively non-blue laccases, which is in accordance with the results shown in Fig. 1. The phylogenetic analysis also revealed a closer evolutionary relationship of rLac-*HhC125* with a partially characterized putative laccase from *B. halodurans* (protein id: AAP5708) (Ruijssenaars and Hartmans 2004; Ferner-Ortner-Bleckmann et al. 2011) described as an analogue of the sequence of *H. halodurans* C-125 laccase (protein id: BAB64332) (Fig. 2a).

Ruijssenaars and Hartmans (Ruijssenaars and Hartmans 2004) did not provide details on *B. halodurans* laccase expressed as a recombinant protein in the *E. coli* system with only a few variables studied, including optimal values of pH and temperature. In the present work, the laccase of *H. halodurans* C-125 was expressed as a derivative protein lacking the signal peptide to avoid the exportation of the recombinant enzyme into different fractions (i.e., cytoplasmic, periplasmic, and extracellular), leading to a weak protein expression recovery. In this study, we did not carry out the production of a crude laccase from *H. halodurans* C-125. We used the recombinant enzyme technology, owing the fact that fermentation of *H. halodurans* leads to the production of multiple types or isoforms of the enzyme that may differ in their stability, catalytic efficiency, activity on substrates, and period of incubation. The recombinant enzyme technology used in this study allowed us to isolate and clone the gene encoding the specific oxidase of interest into the prokaryotic expression vector (plasmid DNA). Multicopy plasmid DNA (pDNA) is a promising molecule for increasing the protein production yield. Such strains as *E. coli* BL21 (DE3) are easily cultured with simple nutritional requirements, exhibit fast growth kinetics (doubling time of about 20 min), and are easily transformed with exogenous DNA.

An about 1434-nucleotide-long fragment was PCR amplified with primers containing engineered restriction sites for direct cloning into the promoter-based prokaryotic expression vector T7/lac (Fig. 2b). Thus, the amplified gene was digested with *Bam*HI and *Hind*III and cloned into pET28-a ( +). The recombinant plasmid was first maintained in *E. coli* DH5α for vector propagation and then checked by colony PCR (data not shown). The presence of the laccase gene was confirmed by plasmid sequencing (data not shown). The DNA sequencing of the pET28-a ( +)/*Lac* revealed the correct insertion of the *Lac* gene, and the corresponding enzyme was designated as rLac-*HhC125*.

The visualization of the 3D structure model with the PyMol tool revealed that rLac-HhC125 is a monomeric protein and possesses three domains (Fig. 2c). About 100% of the rLac-HhC125 residues were modelled with the I-Tasser tool with C-score = -0.67, TM score = 0.603 ± 0.14 and RMSD = 8.7 ± 4.6 Å. The primary domain (represented in green colour in Fig. 2c) of the rLac-HhC125, which makes the *N-terminal* part, possesses 7 filaments arranged in a β-barrel shape. Domain 2 (shown in yellow in Fig. 2c) of the rLac-HhC125 possesses a β-barrel made up of 6 filaments. At the end, the *C-terminal* domain of the rLac-HhC125 (pink colour in Fig. 2c) is composed of 9 filaments arranged in a β-barrel type and possesses the attachment site of the substrate at residue Cys480.

### Heterologous expression, optimisation, and partial purification

**Fig. 3:**
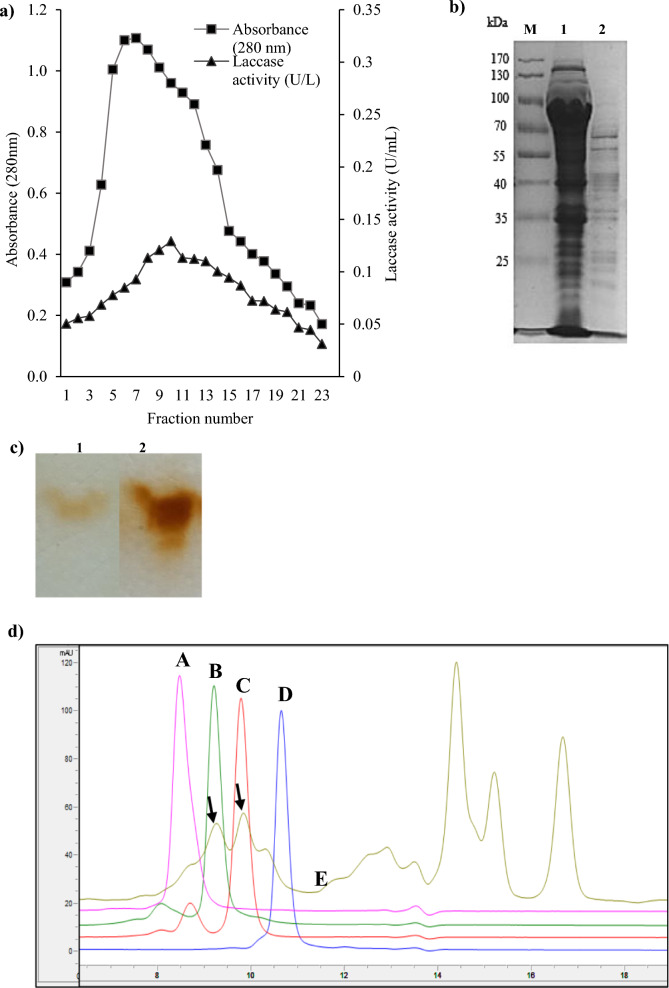
Heterologous expression and purification of putative laccase from *H. halodurans* C-125: **a** Profile of the size exclusion chromatography of a crude laccase sample (Sephadex-G100); elution flow rate: 0.25 mL min^−1^; volume of collected fractions: 1 mL; square symbols (■)—protein concentrations; triangle symbols (▲)—laccase activities using SGZ (substrate) before the ultrafiltration; **b** 12% SDS-PAGE analysis; M—protein ladder; lane 1—total protein extract; lane 2—rLac-*HhC125* laccase sample after SEC; **c** Zymogram analysis of laccase activity using guaiacol as a substrate; lane 1—rLac-*HhC125* laccase sample after SEC, lane 2—*C. unicolor* purified laccase; **d** SEC-HPLC analysis of protein standards 158 kDa (**A**), 75 kDa (**B**), 43 kDa (**C**), 29 kDa (**D**), and rLac-*HhC125* laccase sample (**E**) with the observed two peaks assigned as the laccase protein (marked with arrows)

Recombinant laccase referred as rLac-*HhC125* was produced as a soluble enzyme lacking the native signal peptide sequence for the intracellular expression in *E. coli* as an expression host. For successful heterologous gene expression in *E. coli* BL21(DE3), we tested whether the supplementation of Cu^2+^ ions in the expression TB medium would improve the laccase transcription and the laccase activity. Thus, the rLac-*HhC125* culture medium was enriched with 2 mM CuSO_4_ with a varying concentration of IPTG to avoid a toxic effect on cell growth. We found that the recombinant laccase was optimally expressed when the transcription of the laccase gene was established with 0.5 mM IPTG and 2 mM CuSO_4_ synergy at 19 °C and a shaking speed of 190 rpm for 18 h. The laccase was produced with an estimated specific activity of 0.2 U mg^−1^ using SGZ as a substrate (Table 1). The peak showing laccase activity was eluted at 0.02 M sodium phosphate, pH 7 (Fig. 3a). Fractions containing major laccase activity were pooled and concentrated through ultrafiltration (10 kDa cut-off) to obtain a partially purified laccase with 57.6% yield and an estimated specific activity of 0.3 U mg^−1^. This enzyme was used for the thorough characterisation of the *H. halodurans* C-125 laccase. The SDS-PAGE of *H. halodurans* C-125 laccase sample after SEC chromatography (Fig. 3b) did not show only a single band corresponding to the molecular mass of that calculated using the Protparam tool (http://web.expasy.org/protparam/). The theoretical molecular weight of the rLac-*HhC125* monomer calculated in this way is 54 kDa. The electrophoresis of the partially purified enzyme and substrate-staining revealed that BL21(DE3) harbouring plasmid pET-28(a) + /Lac produced one band displaying laccase activity which showed the similarity to laccase isolated from the fungus *Cerrena unicolor* (Fig. 3c). The laccase protein spots in *C. unicolor* were observed between 35 and 70 kDa among them to 55 kDa isoform (Pawlik et al. 2021). The SEC-HPLC profile of the rLac-*HhC125* enzyme sample revealed 2 peaks corresponding to the molecular weights of 43 kDa and 75 kDa (Fig. 3d). These two peaks might correspond to the monomer and dimer form of the studied laccase.

**Table 1:** Oxidation of syringaldazine (SGZ) by rLac-*HhC125*

Substrate	Formula	Reading wavelength (nm)	pH value	Cofactor (CuSO_4_)	Absorbance (ΔA min^−1^)	Activity ± SD (U mL^−1^)
SGZ		530	8	2 mM	0.46 ± 0.005	0.36 ± 0.004

### Enzymatic activity optimization and biochemical characterization

The biochemical properties were studied using a partially purified laccase from the recombinant BL21(DE3) carrying pET28-a ( +)/*Lac*. All the measurements necessary for the characterization were carried out in triplicates, and the results are represented as mean ± standard deviation.

### Laccase activation by the presence of copper and thermal pre-treatment

**Fig. 4:**
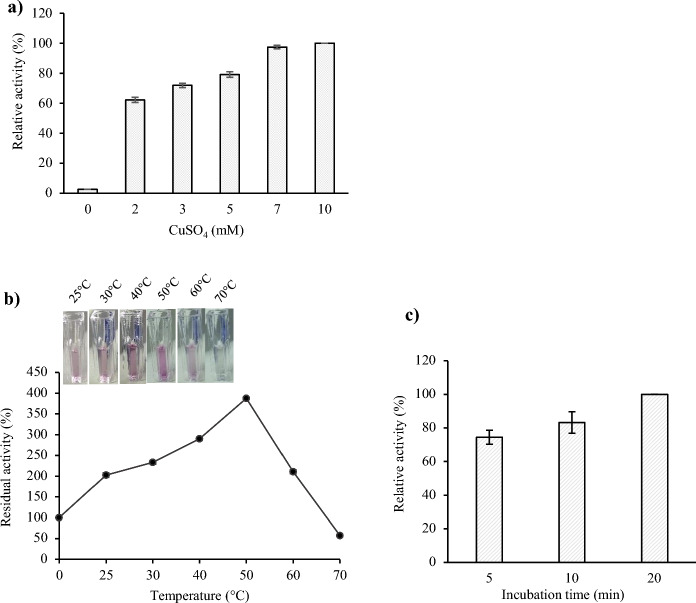
Optimisation of the pre-treatment step for standard assay conditions for assessment of the activity of partially purified rLac-*HhC125*: **a** determination of the optimal Cu^2+^ concentration; **b** estimation of the temperature for 20 min; **c** estimation of the incubation time

No SGZ oxidation activity was detected in the assay solution composed only of buffer (pH 8) and recombinant *H. halodurans* C-125 laccase. Thus, we analyzed the influence of CuSO_4_ used as a cofactor and the thermal-activation pre-treatment step on the enzymatic activity. The influence of CuSO_4_ added to the reaction mixture in different concentrations (1–10 mM) was investigated using SGZ as a more specific substrate for partially purified laccase. As shown in Fig. 4a, a significant increase in laccase activity along with the increasing amount of CuSO_4_ (1 to 10 mM) was observed (Fig. 4a). However, the higher concentration of CuSO_4_ in the assay solution (from 7 mM of CuSO_4_) led to a precipitate generation (data not shown); therefore, the 2 mM concentration was selected as a sufficient amount to activate laccase. Based on our results, we demonstrated the importance of Cu^2+^ for the rLac-*HhC125* activity. These results indicate that this bacterial laccase extracted from induced BL21 (DE3) *E. coli* cells may occur as an apoenzyme (inactivated form) and requires the supply of copper for the reaction. The copper-dependence of rLac-*HhC125* is consistent with previous studies on laccases extracted from *B. subtilis* WD23 (Wang et al. 2016), *B. licheniformis* NS2324 (Chopra and Sondhi 2022), and a microbial consortium WSC-6 (Zhang et al. 2021). One possibility of the synthesis of a copper-depleted laccase is the incomplete incorporation of Cu^2+^ ions from the culture media. In previous reports, *E. coli* was identified to process two copper ion homeostasis systems (*Cue* and *Cus* systems), which catalyze the removal of excess copper ions from cells, maintaining a balanced copper concentration in the bacterial cytoplasm (Gunne et al. 2013; Safary et al. 2016). To overcome this problem, the change in aeration from shaking (aerobic) to stationary (anaerobic) conditions enhances the copper content in cultivated cells (Durão et al. 2008). Since Outten and co-workers showed the increased toxicity of copper in anaerobic conditions (Outten et al. 2001), a culture in aerobic conditions and an in vitro copper reconstitution of soluble rLac-*HhC125* during the assay were performed to obtain a holoenzyme (active form). Thus, CuSO_4_ was added to the buffer solution in the final concentration of 2 mM CuSO_4_ for all subsequent enzyme activity determination tests (standard assay conditions).

Temperature-dependent rLac-*HhC125* activation was shown (Fig. 4b). The stimulation of laccase activity by temperature was already observed at 25 °C with an approximately twofold increase in laccase activity and its maximum at 50 °C with a fourfold increase, compared to its activity in the original non-thermally activated laccase sample. Our observation is similar to previous literature reports, including those on *Bacillus* laccase (Mollania et al. 2018) and fungal laccase (Yang et al. 2020). The further rise in the temperature during the preincubation step caused a loss of enzyme activity. Hence, it can be assumed that the thermal treatment of the rLac*HhC125* at 50 °C for 20 min before the assay promotes its structure flexibility and has a role in the conversion of the enzyme molecules into their active form and possible incorporation of copper into the active centre (Yang et al. 2020). Moreover, the incubation time at 50 °C was optimized and represented in Fig. 4c.

### Activity and stability of recombinant laccase in different pH and temperature conditions

**Fig. 5:**
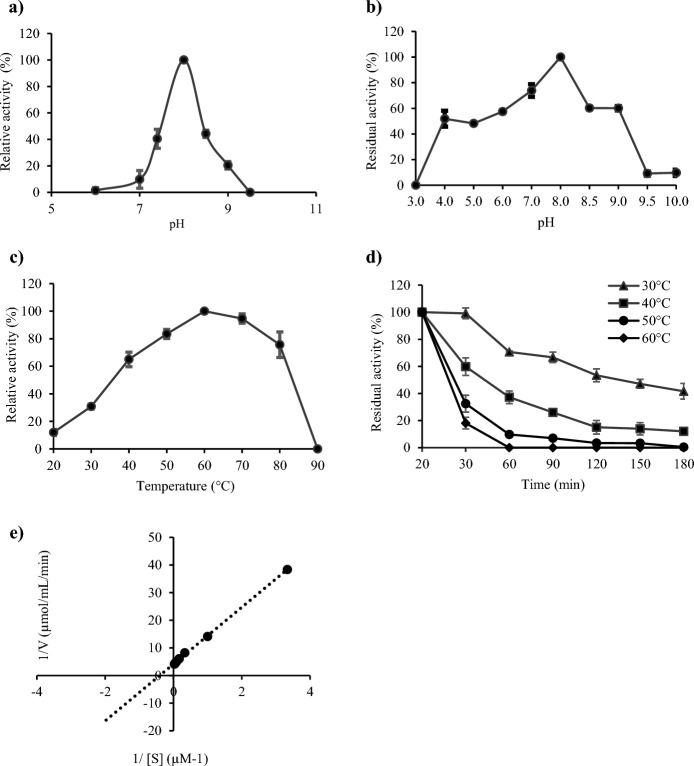
Biochemical characterization of partially purified rLac-*HhC125* using SGZ as a substrate: **a** optimal pH value for the activity; **b** stability for 6 h at different pH values; **c** optimal temperature for the activity at pH 8.0; **d** thermal stability for 180 min at different temperatures (30 °C, 40 °C, 50 °C, 60 °C); **e** Lineweaver–Burk double reciprocal plots of the SGZ concentration versus laccase activity for determination of the *K*_M_ value

The optimal pH values for the activities of fungal laccases are usually in the acidic pH range (Loi et al. 2021). However, some industrial bioprocesses based on such oxidoreductases as laccases may require neutral to alkaline pH values, which increases the range of their possible applications. The recombinant laccase rLac-*HhC125* presented in this study exhibited a bell-shaped profile with maximum activity at pH 8 using SGZ as a substrate in the standard assay conditions and with a concomitant lack of activity at pH 6 and 9.5 (Fig. 5a). The maximum activity of the rLac-*HhC125* observed at alkaline pH using SZG as a substrate is in accordance with previously reported crude bacterial laccases from *Bacillus* sp. WT (Siroosi et al. 2016), *B. pumilus* W3 (Guan et al. 2014), *S. coelicolor* A3(2) (Yadav et al. 2018), and *Thermus thermophilus* (Liu et al. 2015). Noteworthy, some described recombinant bacterial laccases exhibited a neutral (Brander et al. 2014; 2019) or acidic range of the optimum pH (Pawlik et al. 2016). The pH stability of the rLac-*HhC125* was determined at the range of pH between 3 and 10 using different types of buffers, which is summarised in Fig. 5b. Laccase retained 100% activity after 6 h of incubation at pH 8 and found to be most stable at this pH value. In addition, the rLac-*HhC125* maintained around 60% of its maximum activity at more alkaline pH values (8.5–9) and over 50% at a weakly acidic pH value (6). Similar results were obtained for *B. pumilus* W3 (Guan et al. 2014) and *B. licheniformis* (Lu et al. 2013), which exhibited 100% stability at alkaline pH.

The optimal temperature for the activity of the partially purified laccase was 60 °C (Fig. 5c), which was higher than that of *B. coagulans* LMCO (Ihssen et al. 2015), *B. subtilis* R5 (Basheer et al. 2019)*, B. pumilus* W3 (Guan et al. 2014), and *Bacillus sp*. WT (Siroosi et al. 2016). In the case of *B. licheniformis* (Lu et al. 2013)*,* laccase demonstrated an optimum temperature at 70 °C. In our study, laccase activity started to decrease at 70 °C, maintaining 95% of its maximum activity. The further increase in the temperature resulted in laccase denaturation at 90 °C. The analysis of the thermal stability of the partially purified laccase was conducted in the range between 30 °C and 60 °C for 180 min (Fig. 5d). The rLac-*HhC125* was found to be very stable after 30 min of incubation at 30 °C and 40 °C, retaining 99% and 60% of its maximum activity, respectively. The incubation of the rLac-*HhC125* for 90 min at 30 °C resulted in loss of activity to 66.7%, whereas at 40 °C, this recombinant laccase maintained only 26% of its catalytic activity. The increase in the temperature resulted in a decrease in the enzyme stability. The preincubation at 50 °C and 60 °C for 30 min yielded residual activity of about 32.5% and 18.1%, respectively. The treatments of the rLac-*HhC125* at 30 °C and 40 °C for 180 min resulted in a sharp decrease in the initial activity to zero in the temperature range of 50–60 °C. Based on the thermal stability assays, the rLac-*HhC125* half-life (T_1/2_) was 120 min at 30 °C, 45 min at 40 °C, 25 min at 50 °C, and 25 min at 60 °C, using SGZ as a substrate. The moderate rLac-*HhC125* stability in the range of 30–40 °C was a shortcoming. The apparent *K*_M_ (*K*_M (app)_) value for SGZ in the standard conditions was estimated to be 2.48 μM (Fig. 5e). This is comparable to other recombinant laccases, such as the laccase from the *B. subtilis* endospore (CotA) obtained from *E. coli* as a host and the laccase from *B. licheniformis* expressed in the *Picha pastoris* host with the *K*_M (app)_ values of 4.1 μM and 26 μM respectively (Martins et al. 2002; Lu et al. 2013).

### Effect of metal ions

The resistance of laccase to adverse environmental conditions has attracted considerable attention due to its potential to be used for environmental remediation and in industry. Because metal ions are present in many environmental contaminants, laccases that are more resistant to metal ions are desirable (Yang et al. 2020). The presence of Mn^2+^, Mg^2+^, Fe^2+^, or Zn^2+^ ions (1 mM final concentration) had different effects on the rLac-*HhC125* activity measured directly after their addition to the mixture of the pre-treated laccase solution after 20-min incubation in the standard assay conditions (20-min incubation at 50 °C). The tested metals showed two types of behaviour. When the Mn^2+^ ions were added to the assay solution, we observed a slight decrease in SGZ oxidation. This activity decreased 2.5-fold after the laccase pre-treatment with Mn^2+^ ions for 20 min, suggesting that this ion did not have an impact on the substrate itself but acted on the laccase structure. Moreover, the Zn^2+^ and Fe^2+^ ions exerted either strong inhibitory effects on the catalysis of the substrate (competitive inhibition) or a destructive effect on the laccase structure (Table 2). Similar results were observed in *B. licheniformis* NS2324 (Chopra and Sondhi 2022) and *Streptomyces* sp. SB086 (Fernandes et al. 2014). By contrast, the Mg^2+^ ions promoted the laccase activity (1.2-fold increase) when the rLac-*HhC125* was pre-treated for 20 min. The promoting effect of MgSO_4_ was in line with previous research (Fernandes et al. 2014).

**Table 2:** Influence of metal ions (1 mM), inhibitors (1 mM), and organic solvents (10%) on the rLac-*HhC125* residual activity measured in kinetic tests immediately after the addition of the tested compound (0 min) and after 20 min of exposure (20 min) during the laccase pre-treatment step in the standard assay conditions using SGZ as the substrate

	Residual activity (%) ± SD (0 min)	Residual activity (%) ± SD (20 min)
Control/metal ions		
Control	100*	100*
Mg^2+^ (MgSO_4_)	101.8 ± 8.8	122.7 ± 10.1
Mn^2+^ (MnSO_4_)	89.8 ± 11.2	40.99 ± 3.6
Zn^2+^ (ZnSO_4_)	13.8 ± 8.2	11.5 ± 3.6
Fe^+^ (FeSO_4_)	8.78 ± 2.8	6.65 ± 0.77
Control/inhibitors		
Control	100*	100*
EDTA	84.7 ± 4.6	84.9 ± 7.1
Sodium azide	64.2 ± 3	64.7 ± 5.8
L-cysteine	0	ND
Phenylthiourea	3.9 ± 3.8	ND
Control/ organic solvents		
Control	100*	100*
Methanol	101.3 ± 8.7	94.6 ± 9.3
Ethanol	110.7 ± 12.1	107.5 ± 1.6
DMSO	84.1 ± 3.1	91.3 ± 3.7
Acetonitrile	177.2 ± 19.6	201.1 ± 15.8

SD: Standard Deviation; ND: Non-Determined; *control—the SGZ oxidation in the standard laccase assay conditions and without metal ions (inhibitors or organic solvents) was recorded as 100%

### Effect of inhibitors

The inhibition of enzymes is an important topic for controlling environmentally friendly biocatalytic industrial processes. Therefore, we studied the effect of different inhibitors (1 mM final concentration) on SGZ oxidation immediately after the addition of the inhibitor (time 0) and after 20-min incubation with laccase in the standard assay conditions. The effect of inhibitors on the catalytic oxidation activity against SGZ is represented as a percentage concerning the control laccase assayed in the standard assay conditions (100%) (Table 2). The results showed high stability of the laccase in the presence of EDTA (84.86%) and sodium azide (64%) regardless of the incubation time. The presence of the other tested inhibitors, such as phenylthiourea and L-cysteine, caused a decrease in laccase activity to a value of 3.9% and 0% (time 0), respectively. The resistance towards the EDTA chelator may be ascribed to the low accessibility of EDTA to the structural copper atoms of the recombinant rLac-*HhC125* active center (Safary et al. 2016). The tolerance to EDTA was also reported in *B. licheniformis* LS04 (Lu et al. 2013). However, *B. licheniformis* O12 laccase was reported to be inhibited by EDTA (Kesebir et al. 2021). The strong inhibitory effect of L-cysteine at 1 mM is in line with that exhibited by other laccases from *B. licheniformis* LS04 (Lu et al. 2013) and *B. subtilis* R5 (Basheer et al. 2019). The total inhibition of rLac-*HhC125* activity in the presence of phenylthiourea was expected, as it is widely used as a control inhibitor (Chaudhary et al. 2021). The decrease in the rLac-*HhC125* activity in the presence of sodium azide was similar to results obtained for *B. tequilensis* SN4 (Sondhi et al. 2014).

### Effect of organic solvents

Organic solvents are often present in biocatalysis, as they increase the solubility of the organic substrates used and thus improve the efficiency of the processes. Therefore, the effect of four commonly used solvents, i.e. methanol, ethanol, acetonitrile, and DMSO, on rLac-*HhC125* activity (0 min) and stability (20 min) was studied in a final concentration of 10%. Based on the results, it can be concluded that ethanol and methanol had a stimulating effect on the recombinant laccase activity and stability (Table 2). Moreover, in the presence of DMSO, the rLac-*HhC125* retained over 84% of its maximum activity. Surprisingly, 10% (v/v) acetonitrile enhanced the laccase activity (approximately 1.77-fold) during the assay (0 min). After 20 min of incubation, the rLac-*HhC125* incubated with acetonitrile showed even higher twofold activity in comparison to laccase which was not treated with the organic solvent. The stimulating effect of organic solvents on laccase activity was observed also in other fungal (Wu et al. 2019; Yang et al. 2020) and bacterial (Lu et al. 2013; Jiang et al. 2021) laccases. Organic solvents also had a negative effect on the activity of some other laccases from *B. licheniformis* O12 (Lu et al. 2013) and *Geobacillus yumthangensis* (Sharma and Leung 2021) by promoting protein unfolding, thereby leading to loss of enzymatic activity. Rasekh et al. reported a correlation between the thermal stability and solvent tolerance of an enzyme (Rasekh et al. 2014), which is in line with the thermal stimulation and solvent tolerance of the rLac-*HhC125* presented in this study.

## Biotransformation of organic compounds as potential dye precursors

**Fig. 6:**
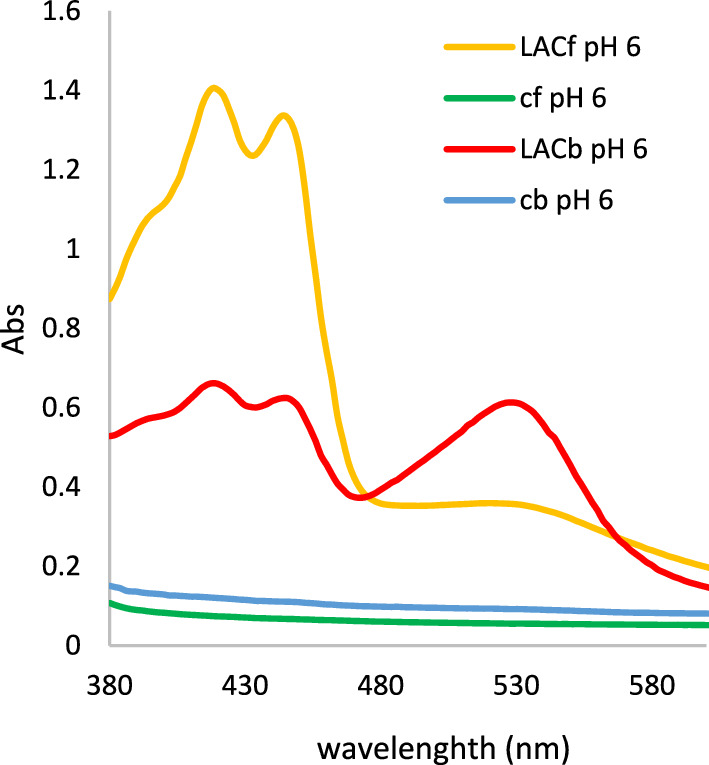
Spectrum of products obtained through the action of bacterial (rLac-*HhC125*) and fungal (LAC) laccase on 2-amino-3-hydroxypyridine at pH 6: *LACf* fungal laccase, *LACb* bacterial laccase, *cb* autoxidation control without bacterial laccase, *cf* autoxidation control without fungal laccase

Laccases have high commercial potential as green catalysts for the transformation of different organic substrates. Among the variety of reactions catalyzed by laccases, there are reactions of degradation of organic pollutants and the synthesis of new hybrid molecules with new properties and new potential applications (Sondhi et al. 2023; Ayodeji et al. 2023). Laccases that are stable in a wide range of pH can be a very useful tool for such reactions due to the possibility of transformation of various organic compounds with different chemical structures, e.g. compounds previously reported by Polak and Jarosz-Wilkolazka (Polak and Jarosz-Wilkolazka 2012). Hence, the preliminary study of the rLac-*HhC125* used as a biocatalyst in the synthesis of coloured compounds was carried out. The recombinant laccase rLac-*HhC125* (10 U L^−1^) was tested for the oxidation of hydroxy derivatives of different organic compounds at two pH values. Based on a qualitative test, we observed that the majority of the tested organic compounds were autoxidized at pH 8.0, irrespective of the presence of the laccase (supplementary Table 1). At pH 6.0, the recombinant rLac-*HhC125* laccase was able to oxidize most of the tested precursors with different organic structures, including benzene, naphthalene, and pyridine hydroxyl-derivatives into yellow, orange, and brown products (supplementary Table 1), and the colours were in most cases similar to the colours of products arising from *C. unicolor* laccase-mediated transformation. In the case of three substrates, i.e. 4A2HBA, 4MxPh, and HNSA, no oxidation mediated by the bacterial and fungal laccases was observed (Table 3). The coloured products obtained in the control samples at pH 8 (without the addition of laccase) were probably caused by non-enzymatic autoxidation of substrates with -OH groups (mainly phenolic derivatives) promoting the lower potential of molecules and deprotonation of hydroxyl groups, which is similar to the action of laccase (Cannatelli and Ragauskas 2017). At the tested pH values, slight autoxidation of amine hydroxy derivatives and D-catechin was observed. Among the benzene derivatives, compounds having amine substituents in the *ortho*- and *para*-position towards the hydroxyl group (5A2HBA and 3A4HBA) were oxidized by the rLac-*HhC125*. The 4A2HBA *meta* isomer of the above-mentioned compounds was not oxidized to the coloured products by the bacterial and fungal laccases. Naphthalene derivatives that possess both amine and hydroxy groups were also oxidized into coloured products by the tested rLac-*HhC125*. Some of them, such as 4AHNS (*ortho*-position) and 4A5HNDSA (*para*-position), were also slightly autoxidized in the control samples without enzyme addition. Very interesting results were obtained in the case of 2-amino-3-hydroxypyridine (2A3HP) transformed into a red-coloured product after the rLac-*HhC125*-mediated transformation, which is in contrast to the yellow product obtained at pH 6 from the same precursor transformed by both the *C. unicolor* fungal laccase presented in this study and the CotA laccase previously described by Sousa and co-workers (Sousa et al. 2018). The detailed analysis of visible spectra of the products obtained indicated that they have two different spectra with different maxima: at 530 nm for the rLac-*HhC125*-mediated transformation product (red) and 420 nm for the fungal laccase-mediated product (yellow). This red-coloured product having maximum absorbance at wavelength 530 nm was also observed in the fungal laccase-mediated transformation product but in very low amounts (Fig. 6).

**Table 3:** Capability of recombinant laccase rLac-*HhC125* for oxidation of selected organic substrates

Substrate		+ rLac-*HhC125*		− rLac-*HhC125*	
Name	Formula	Wavelength (nm)	Abs ± SD	Wavelength (nm)	Abs ± SD
5A2HBA		430	1.53 ± 0.12	430	0.36 ± 0.03
3A4HBA		434	3.66 ± 0.09	426	0.58 ± 0.02
4A2HBA		–	NOx	–	NOx
AHBS		420	2.99 ± 0.02	420	0.36 ± 0.11
Cat		560	0.48 ± 0.07	–	NOx
dHBdSA		454	0.61 ± 0.17	–	NOx
4AHNS		430	1.23 ± 0.35	430	0.96 ± 0.12
6AHNS		456	1.31 ± 0.27	–	NOx
7AHNS		604	0.91 ± 0.11	–	NOx
4MxPh		–	NOx	–	NOx
4A5HNDSA		456	1.77 ± 0.03	456	0.56 ± 0.06
HNSA		–	NOx	–	NOx
DHP-Ala		600	1.53 ± 0.23	600	0.40 ± 0.06
2A3HP		530	0.7 ± 0.13	–	NOx
D-CatN		394	1.91 ± 0.58	394	0.81 ± 0.24

The laccase oxidation was evaluated in shaking conditions (120 rpm) at 40 °C for 24 h in the presence of 2 mM CuSO_4_ in 0.1 M sodium phosphate buffer (pH 6) after preincubation of rLac-*HhC125* at 40 °C for 10 min for enzyme activation. The oxidation of the laccase-free control solution was also investigated (-rLac-*HhC125*). Data shown represent absorbance after 1 h of oxidation

*Abs* Absorbance, *SD* Standard Deviation, *NOx* No Oxidation detected, *+ rLac-HhC125* reaction mixture with rLac-*Hhc125*, − *rLac-HhC125* laccase-free reaction mixture

## Conclusions

*Halalkalibacterium halodurans* C-125 laccase was cloned and expressed in *E. coli* BL21 (DE3) as an apo-type recombinant protein (named rLac-*HhC125*). The active form of the laccase was achieved by a thermal-treatment activation step and copper sulfate (CuSO_4_) supplementation in the assay solution. The laccase was active at high temperatures and alkaline values of pH and exhibited high tolerance to organic solvents. Interestingly, acetonitrile enhanced (twofold) the catalytic activity of the rLac-*HhC125*. Furthermore, the rLac-*HhC125* efficiently transformed different hydroxy derivatives into coloured compounds at pH 6.0. The biochemical properties of the tested laccase from *H. halodurans* C-125 (NCBI accession number BH2082) suggest that it can be a potential green biocatalyst for different biotechnological processes.

## Supplementary Information

Below is the link to the electronic supplementary material.

Supplementary file1 (DOCX 3568 KB)

## Data Availability

All related data and methods are present in this paper. Additional inquiries can be addressed to the corresponding author.
