# Supplementary material for: Biochemical characterization of a recombinant laccase from Halalkalibacterium halodurans C-125 and its application in the biotransformation of organic compounds
Source: Biotechnol Lett. 2024 Oct 28;46(6):1199–218. doi: 10.1007/s10529-024-03532-w (PMC11550293; doi:10.1007/s10529-024-03532-w)
Supplement: Supplementary file 1 — Supplementary file1 (DOCX 3568 KB) [file 10529_2024_3532_MOESM1_ESM.docx]

**Supplementary Fig.1.** Influence of metals, inhibitors, and organic solvents on the activity of rLac-*HhC125*

**Supplementary table 1.** Colour of the product obtained after 1 h and 24 h oxidation of selected organic compounds mediated by rLac-*HhC125* (+rLac) in comparison to colour of the control samples as the result of autoxidation (-rLac). Fungal laccase from *Cerrena unicolor* (+LAC) was used as a control biocatalyst.

| **pH** | **Substrate** | **1 h** | | **24 h** | |
| --- | --- | --- | --- | --- | --- |
|  |  | **- rLac** | **+ rLac** | **-rLac** | **+rLac** |
| **8** | dHBdSA  4A5HNDSA  3A4HBA  4A2HBA  AHBS  6AHNS  7AHNS  4AHNS  2A3HP  CatN  DHP-Ala  HNSA  4MxPh  Cat  5ASA | 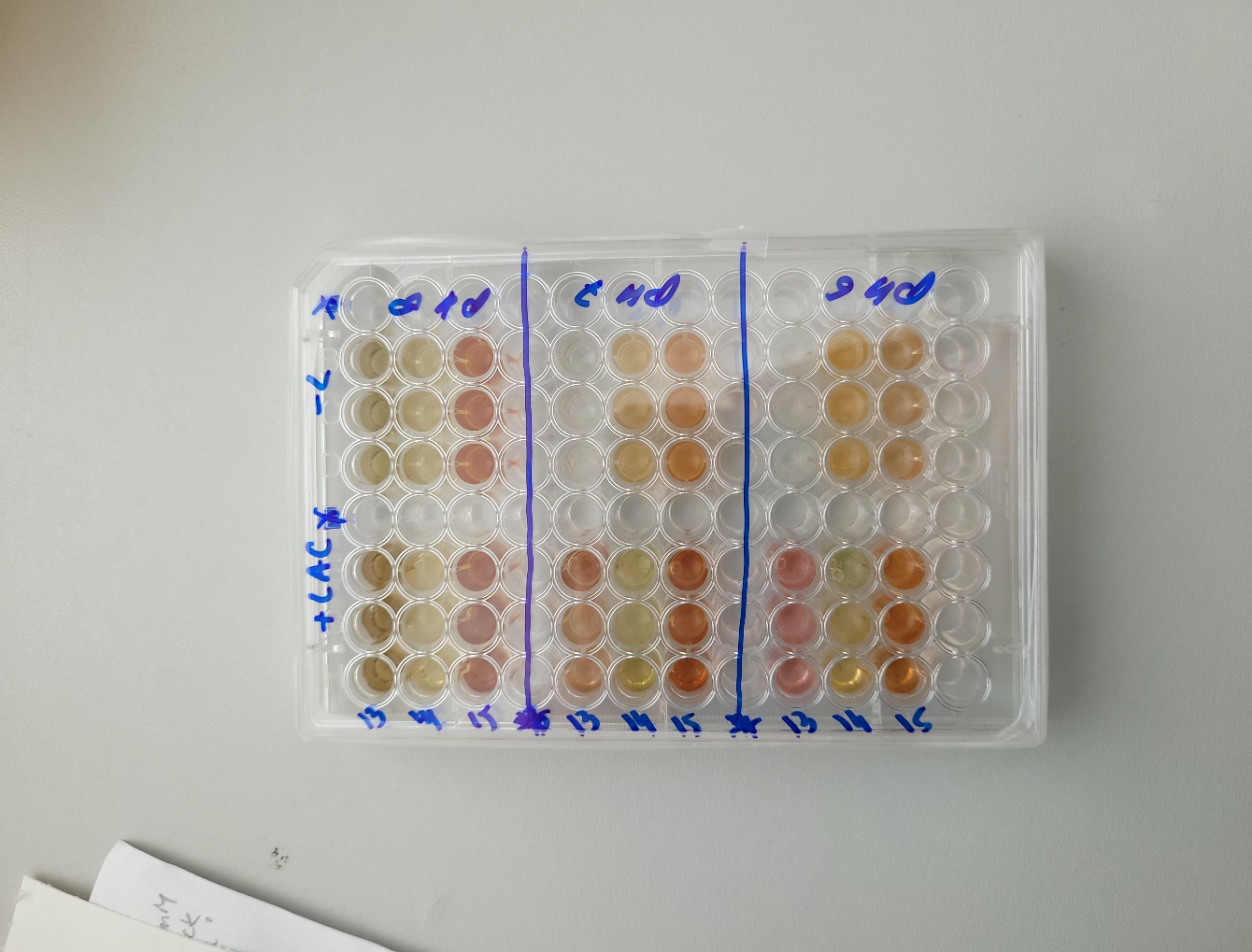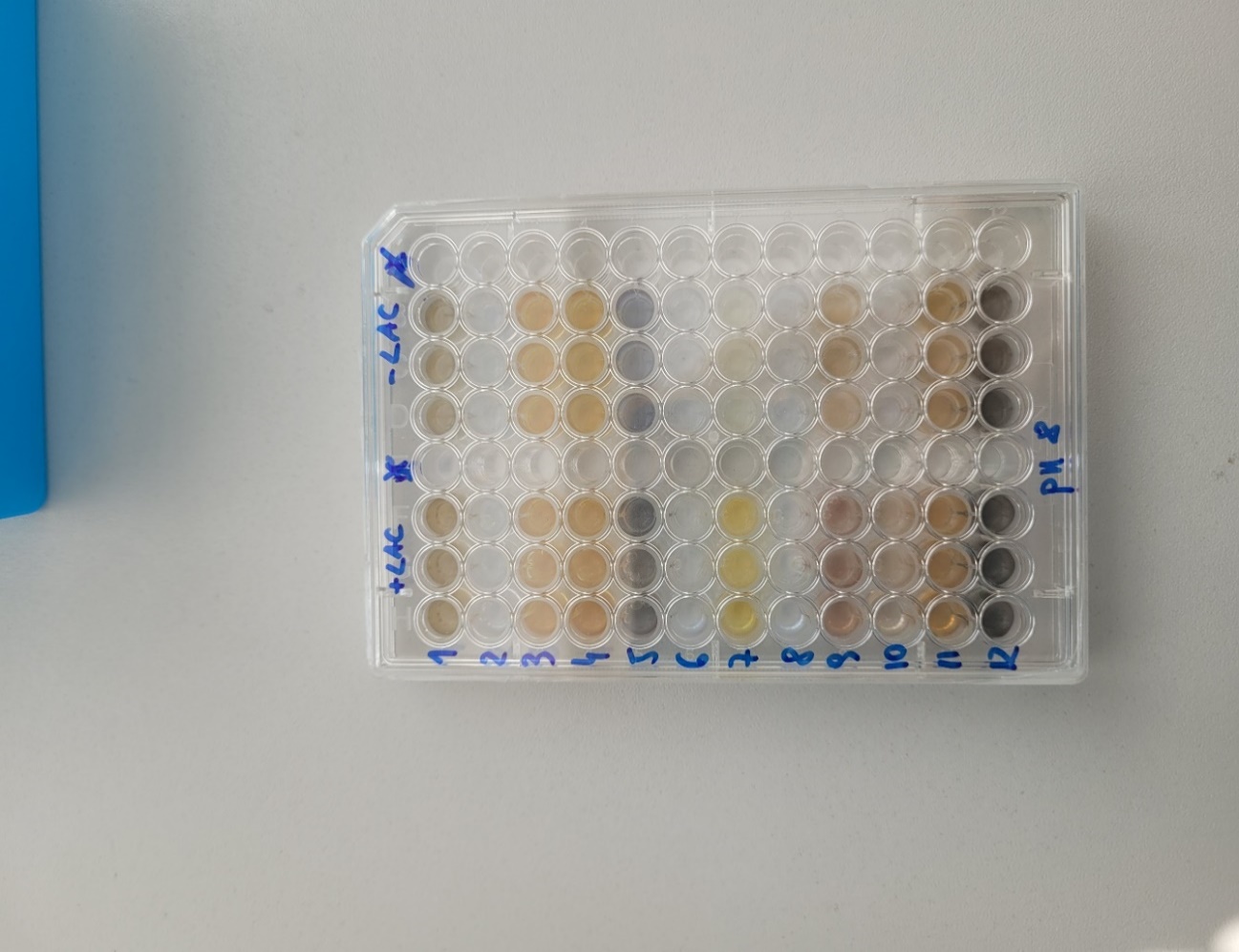 | 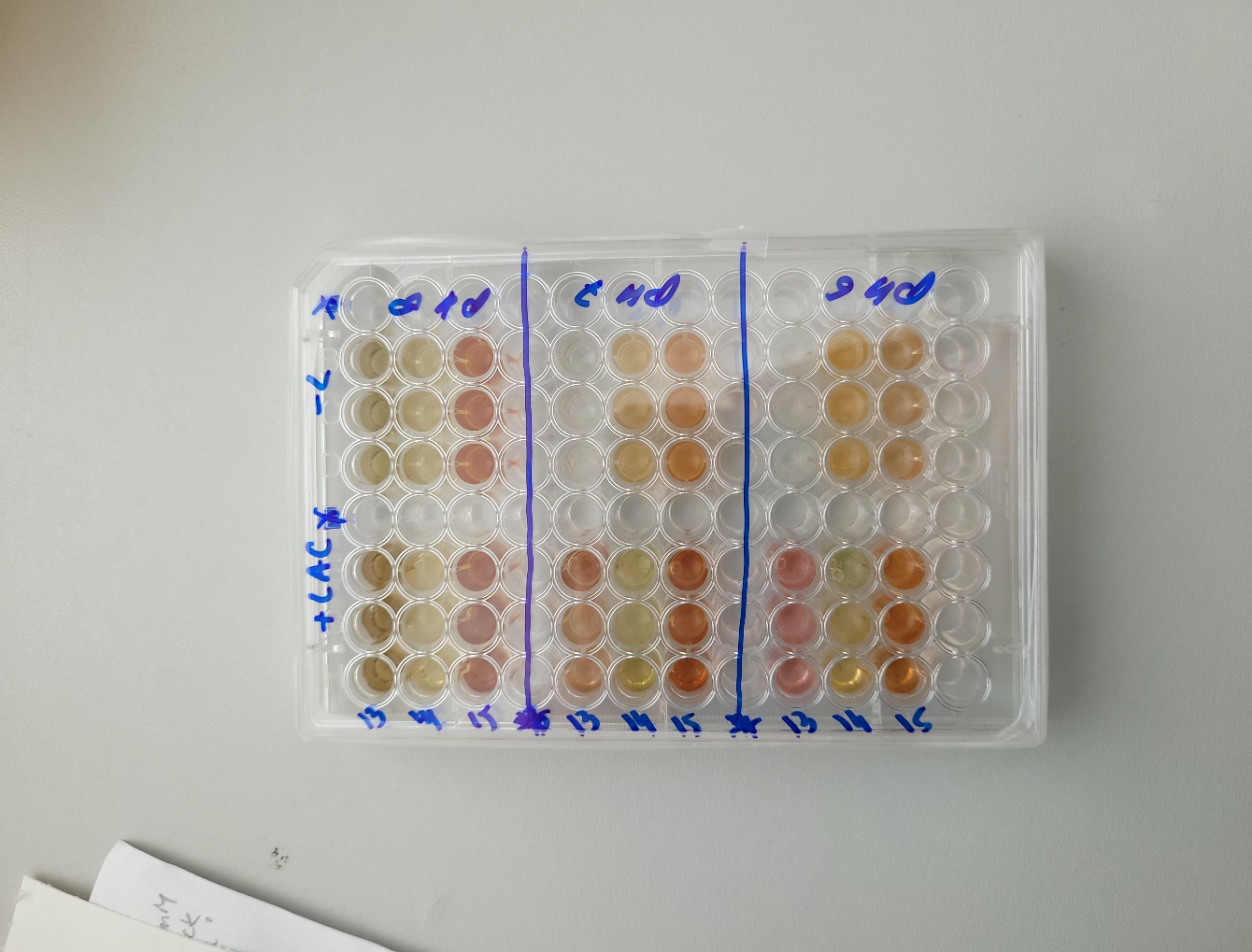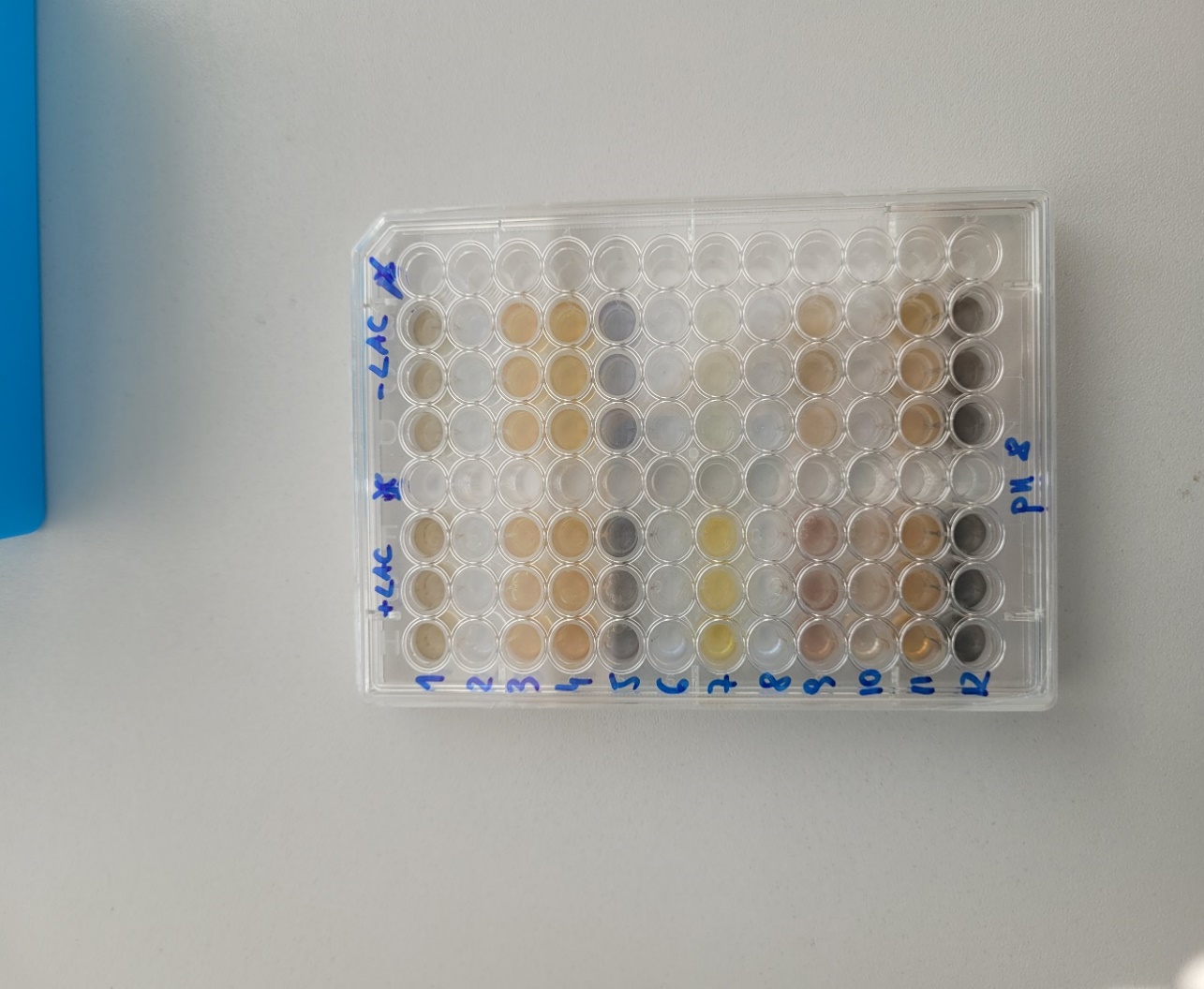 | 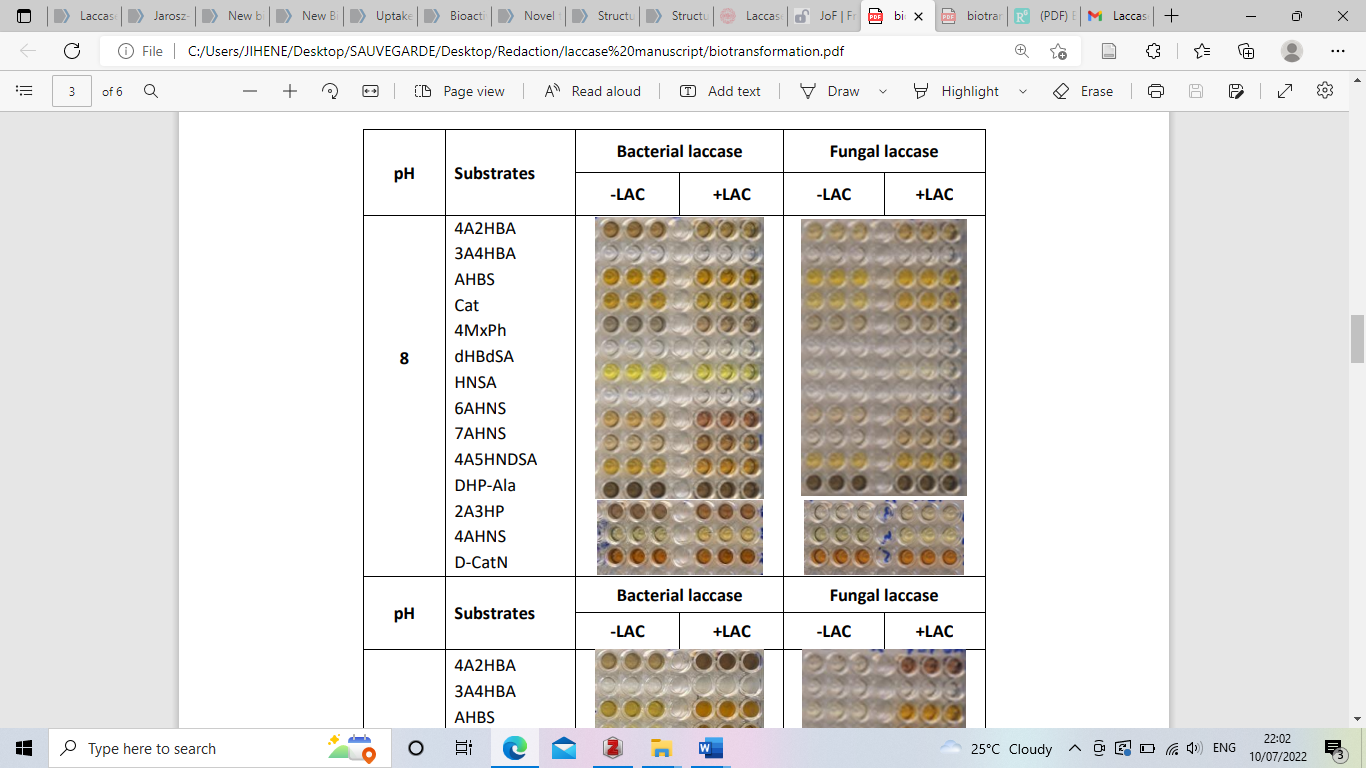 | 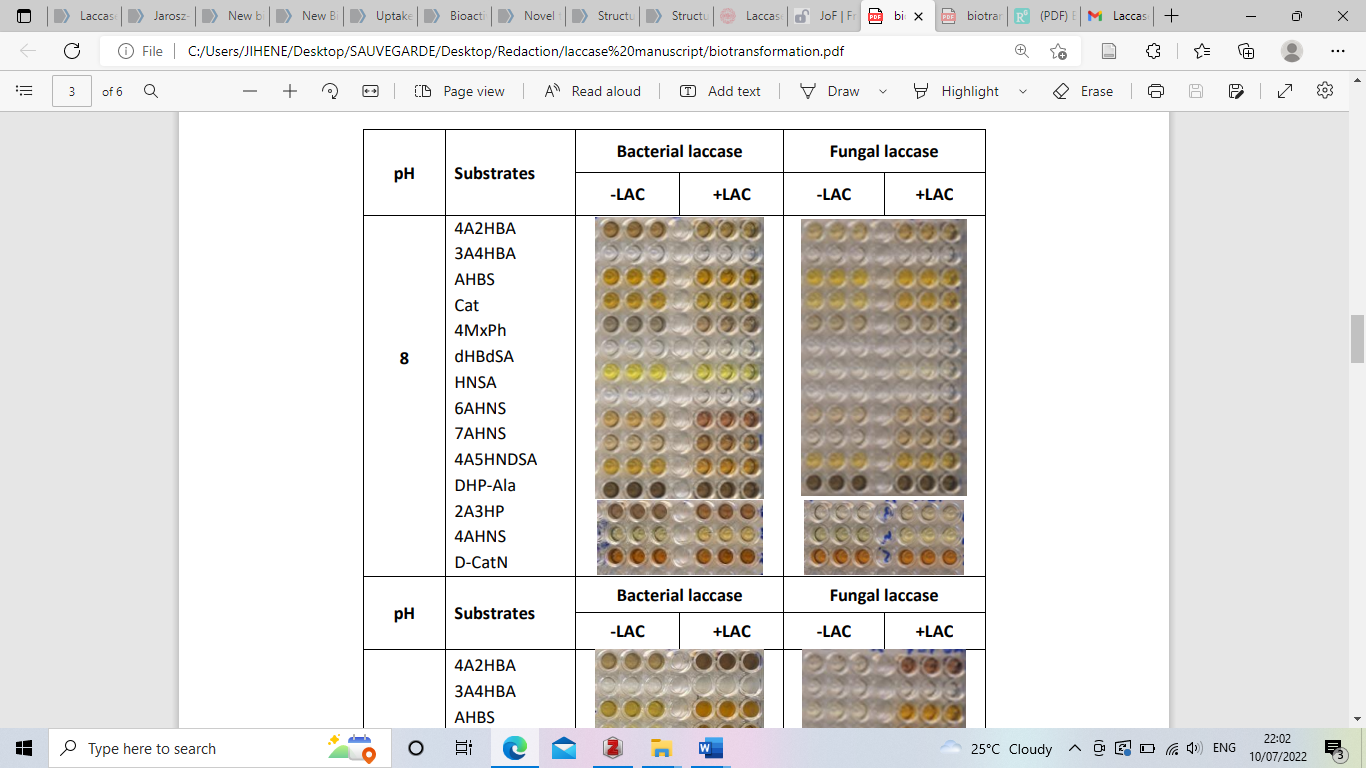 |
| **6** | 4AHNS  2A3HP  4A5HNDSA  7AHNS  6AHNS  dHBdSA  AHBS  4A2HBA  CatN  DHP-Ala  HNSA  4MxPh  Cat  3A4HBA  5ASA | 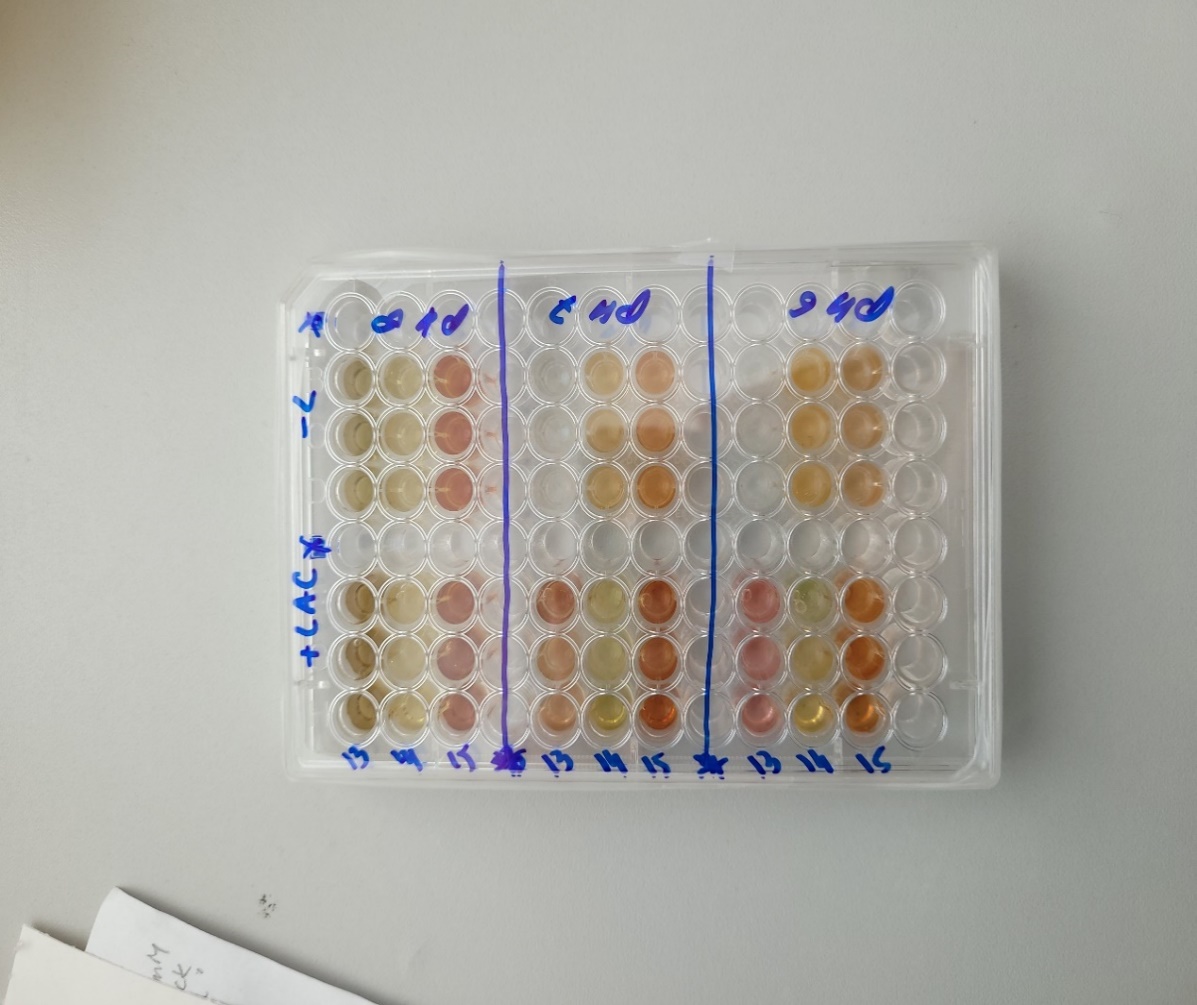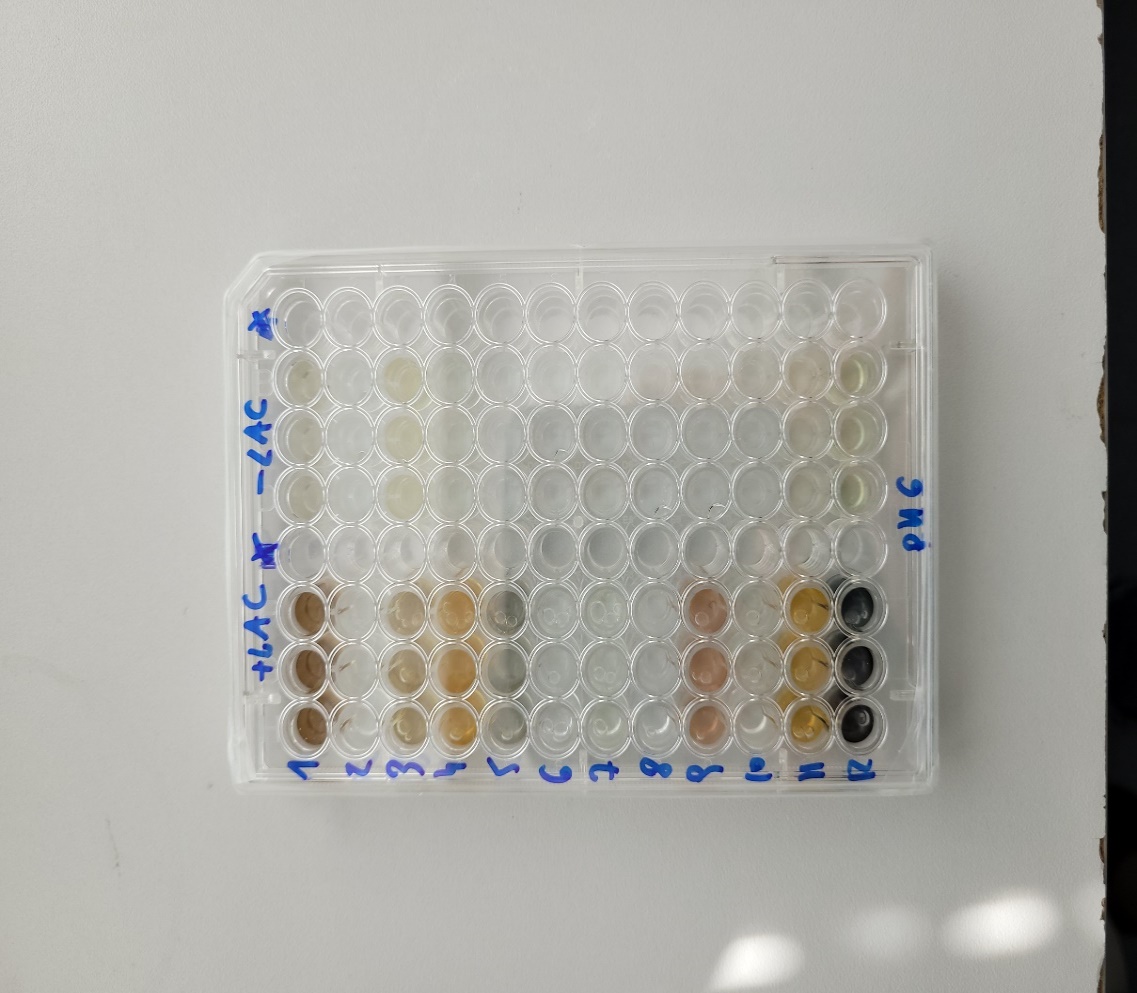 | 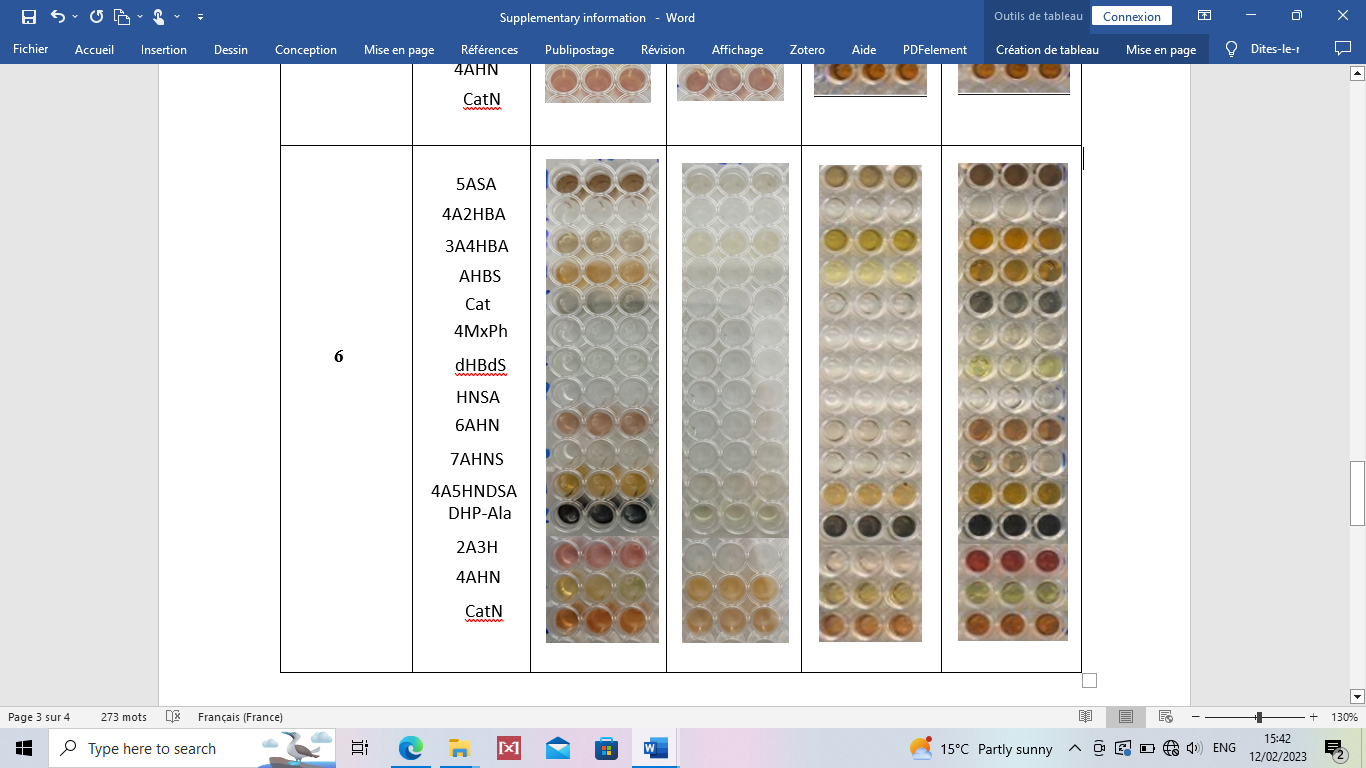 | 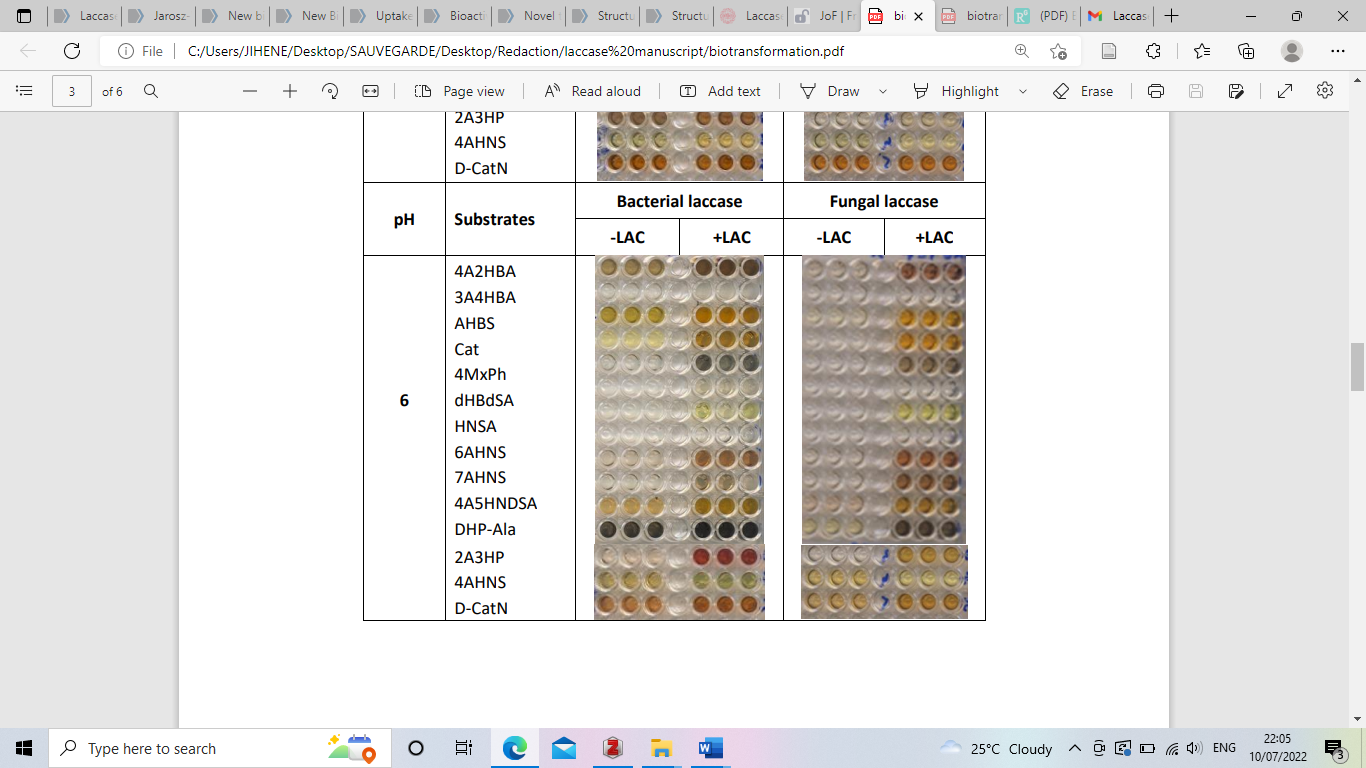 | 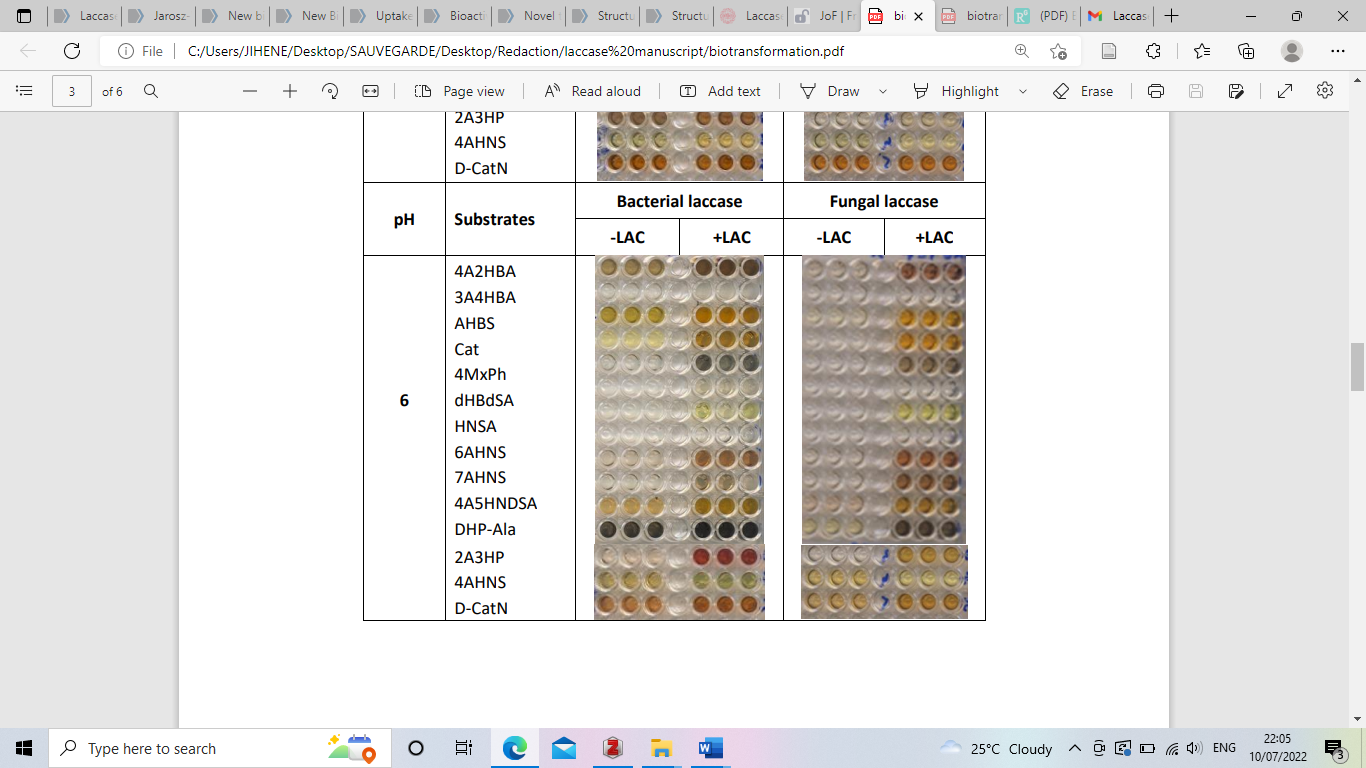 |

| **pH** | **Substrate** | **1 h** | | **24 h** | |
| --- | --- | --- | --- | --- | --- |
|  |  | **-LAC** | **+LAC** | **- LAC** | **+LAC** |
| **8** | 4AHNS  2A3HP  4A5HNDSA  7AHNS  6AHNS  dHBdSA  AHBS  4A2HBA  DHP-Ala  CatN  Cat  4MxPh  HNSA  3A4HBA  5ASA | ND | | 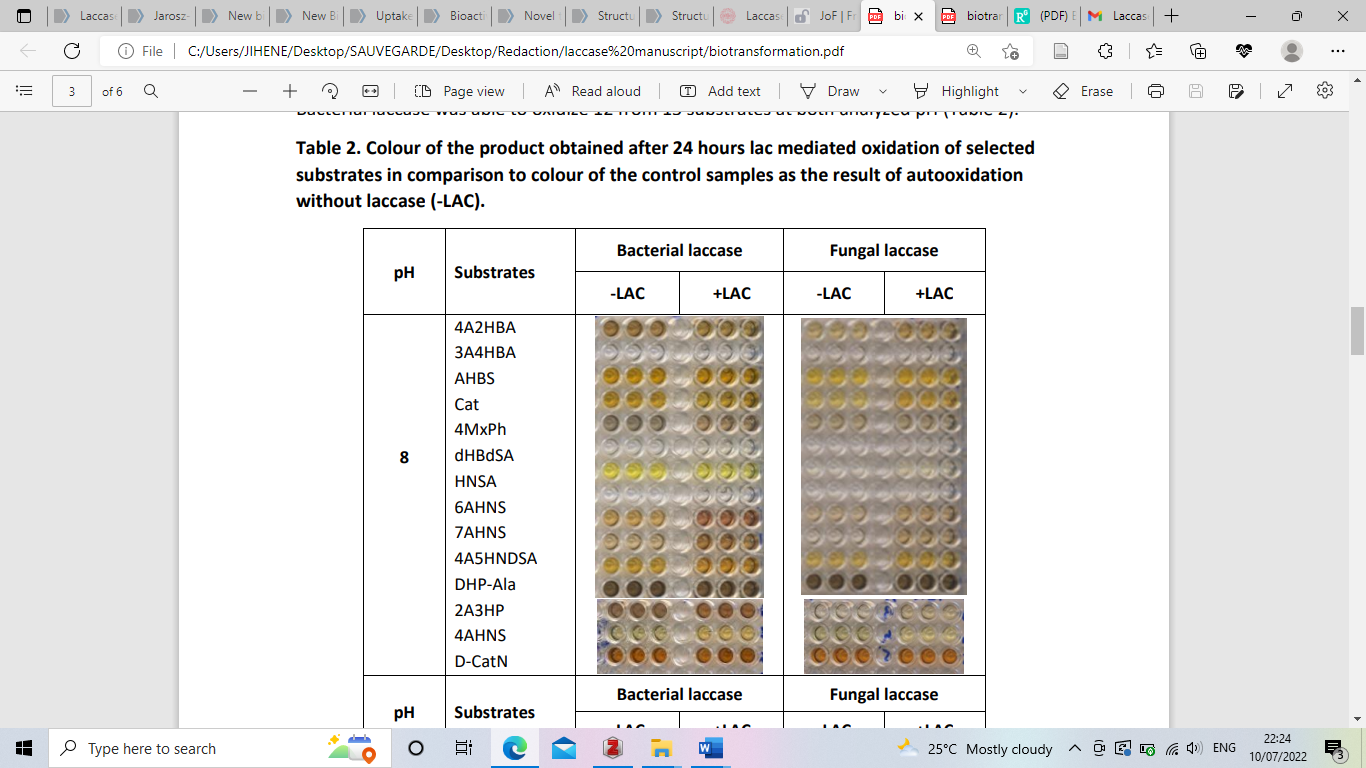 | 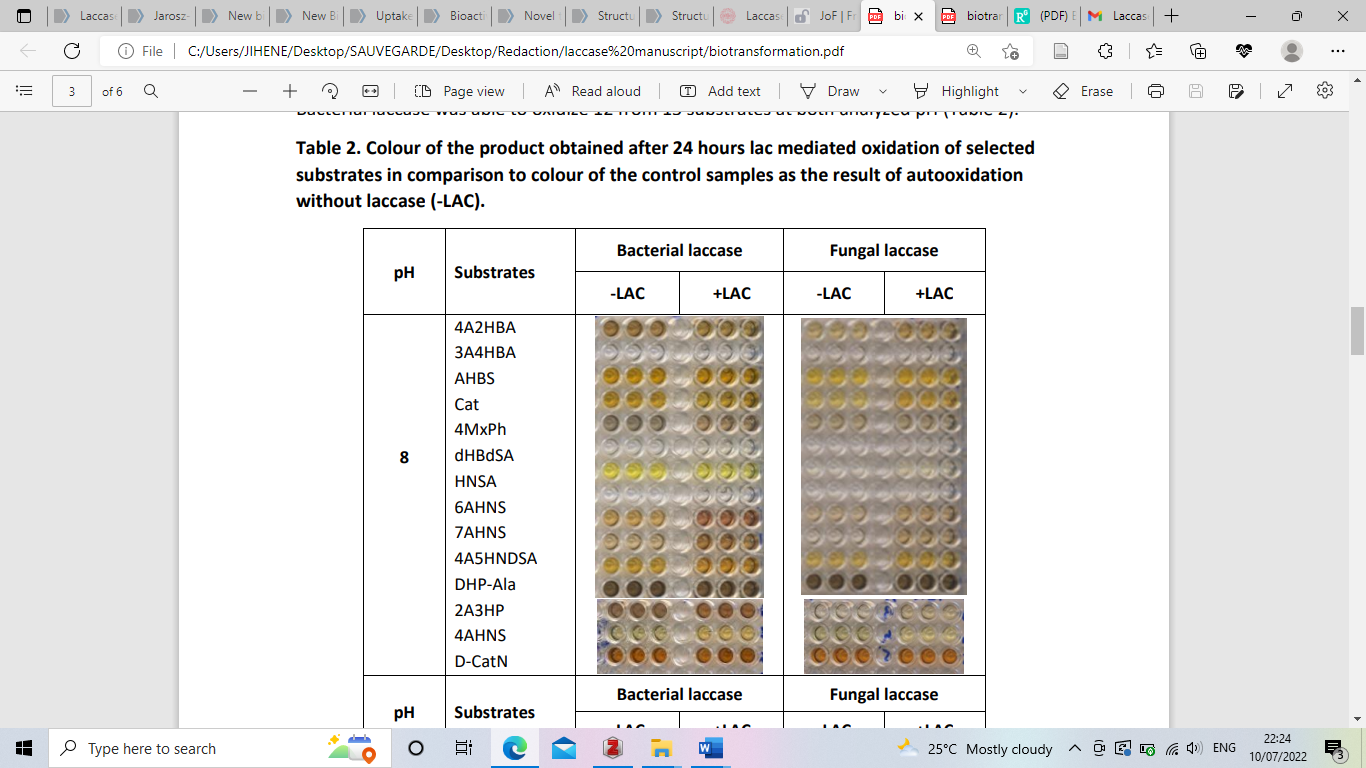 |
| **6** | 4AHNS  2A3HP  4A5HNDSA  7AHNS  6AHNS  dHBdSA  AHBS  4A2HBA  DHP-Ala  4MxPh  3A4HBA  CatN  HNSA  Cat  5ASA | ND | | 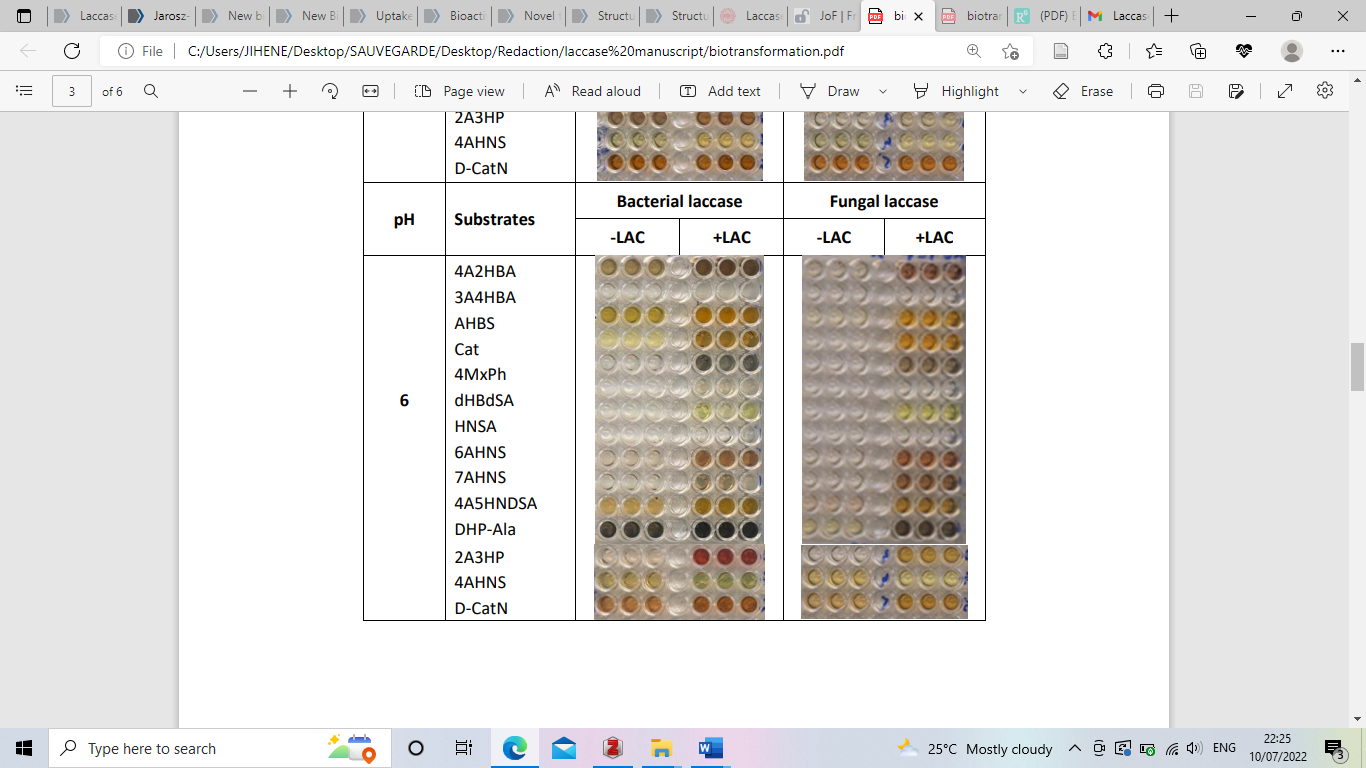 | 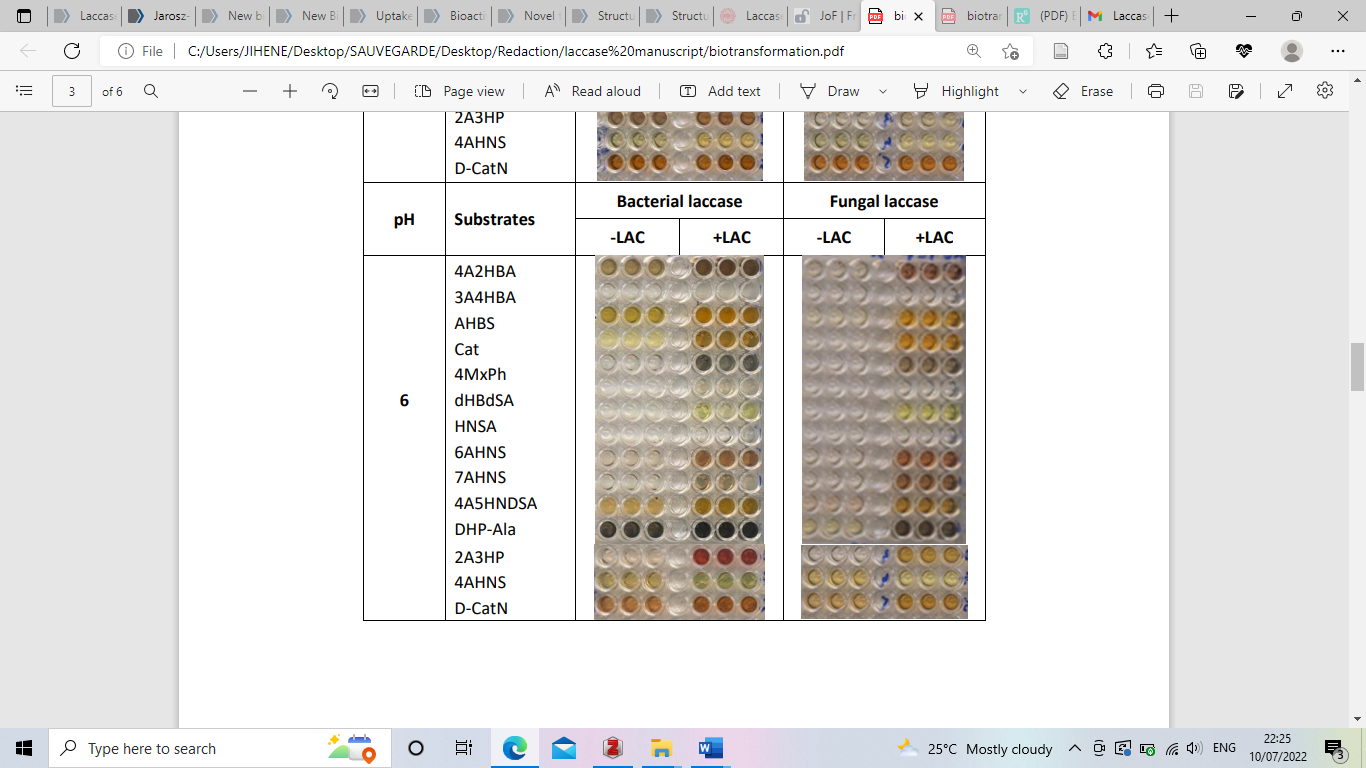 |

**Supplementary table 1.** Continued

_ND: Not Determined._
